# EPRS1-mediated fibroblast activation and mitochondrial dysfunction promote kidney fibrosis

**DOI:** 10.1038/s12276-024-01360-6

**Published:** 2024-12-02

**Authors:** Seung Seob Son, Hee Seul Jeong, Seong-Woo Lee, Eun Soo Lee, Jeong Geon Lee, Ji-Hye Lee, Jawoon Yi, Mi Ju Park, Min Sun Choi, Donghyeong Lee, Sin Young Choi, Jiheon Ha, Jeong Suk Kang, Nam-Jun Cho, Samel Park, Hyo-Wook Gil, Choon Hee Chung, Joon Seok Park, Myung Hee Kim, Jihwan Park, Eun Young Lee

**Affiliations:** 1 Department of Medicine, Graduate School of Soonchunhyang University, Cheonan, Korea; 2 BK21 Four Project, College of Medicine, Soonchunhyang University, Cheonan, Korea; 3 Department of Internal Medicine, Yonsei University Wonju College of Medicine, Wonju, Korea; 4 Research Institute of Metabolism and Inflammation, Yonsei University Wonju College of Medicine, Wonju, Korea; 5 Department of Medicine, College of Medicine, Soonchunhyang University, Cheonan, Korea; 6 Department of Pathology, Soonchunhyang University Cheonan Hospital, Cheonan, Korea; 7 School of Life Sciences, Gwangju Institute of Science and Technology (GIST), Gwangju, Korea; 8 Department of Internal Medicine, Soonchunhyang University Cheonan Hospital, Cheonan, Korea; 9 Institute of Tissue Regeneration, College of Medicine, Soonchunhyang University, Cheonan, Korea; 10 Drug Discovery Center, Daewoong Pharmaceutical Co. Ltd., Yongin, Korea; 11 Microbiome Convergence Research Center, Korea Research Institute of Bioscience and Biotechnology, Daejeon, Korea

**Keywords:** Kidney, End-stage renal disease

## Abstract

Kidney fibrosis causes irreversible structural damage in chronic kidney disease and is characterized by aberrant extracellular matrix (ECM) accumulation. Although glutamyl-prolyl-tRNA synthetase 1 (EPRS1) is a crucial enzyme involved in proline-rich protein synthesis, its role in kidney fibrosis remains unclear. The present study revealed that EPRS1 expression levels were increased in the fibrotic kidneys of patients and mice, especially in fibroblasts and proximal tubular epithelial cells, on the basis of single-cell analysis and immunostaining of fibrotic kidneys. Moreover, C57BL/6 EPRS1^tm1b^ heterozygous knockout (*Eprs1*^+/−^) and pharmacological EPRS1 inhibition with the first-in-class EPRS1 inhibitor DWN12088 protected against kidney fibrosis and dysfunction by preventing fibroblast activation and proximal tubular injury. Interestingly, in vitro assays demonstrated that EPRS1-mediated nontranslational pathways in addition to translational pathways under transforming growth factor β-treated conditions by phosphorylating SMAD family member 3 in fibroblasts and signal transducers and activators of transcription 3 in injured proximal tubules. EPRS1 knockdown and catalytic inhibition suppressed these pathways, preventing fibroblast activation, proliferation, and subsequent collagen production. Additionally, we revealed that EPRS1 caused mitochondrial damage in proximal tubules but that this damage was attenuated by EPRS1 inhibition. Our findings suggest that the EPRS1-mediated ECM accumulation induces kidney fibrosis via fibroblast activation and mitochondrial dysfunction. Therefore, targeting EPRS1 could be a potential therapeutic target for alleviating fibrotic injury in chronic kidney disease.

## EPRS1 inhibition prevents kidney fibrosis and dysfunction

Kidney fibrosis, a common result of chronic kidney disease, leads to irreversible kidney dysfunction. Researchers found that the enzyme EPRS1 plays a key role in this process. Researchers discovered elevated EPRS1 levels in fibrotic kidneys of both patients and mice. The study involved patients, mice, and in vitro cells such as NRK-49F, NIH3T3, and HK-2 cells. The researchers used multiple techniques, including immunohistochemistry, western blot, electron microscopy and single-cell RNA sequencing, to identify EPRS1’s role. They found that EPRS1 promotes fibrosis by activating fibroblasts and causing mitochondrial dysfunction. Single-cell RNA sequencing and western blotting identified the pathophysiological molecular pathways. Inhibiting EPRS1 by genetic and pharmacological methods reduced kidney fibrosis and improved function, suggesting it could be a new treatment for kidney fibrosis. Future research may explore EPRS1 inhibitors as potential therapies for chronic kidney disease.

This summary was initially drafted using artificial intelligence, then revised and fact-checked by the author.

## Introduction

Kidney fibrosis is a common final process in chronic kidney disease (CKD) and is characterized by excessive extracellular matrix (ECM) accumulation^1,2^. Although the wound healing process is necessary for physiological tissue repair and protection of organ function, maladaptive and progressive fibrosis can cause irreversible kidney dysfunction. Fibroblasts and kidney resident cells are stimulated by profibrotic factors such as transforming growth factor β (TGF-β) and connective tissue growth factor (CTGF) under pathological conditions, which serve as major sources of ECM^3–5^. In particular, these factors induce α-smooth muscle actin (α-SMA)-positive differentiation and proliferation of fibroblasts and cause mitochondrial injury in epithelial cells, activating the epithelial‒mesenchymal transition process^6–10^.

Aminoacyl-tRNA synthetase (ARS) is an enzyme that attaches specific amino acids to their corresponding tRNA molecules to form various aminoacyl-tRNAs^11^. Among them, glutamyl-prolyl-tRNA synthetase 1 (EPRS1) is the only bifunctional enzyme with two types of tRNA synthetase (glutamyl-tRNA synthetase 1 or EARS1 and prolyl-tRNA synthetase 1 or PARS1) forming a single structure^12,13^. Since their canonical function is to catalyze the binding of proline to tRNA^Pro^, a major component of collagen, EPRS1-mediated translational regulation of proline-rich proteins is expected to influence the mechanism of fibrosis^14–17^.

EPRS1 has been shown to play a role in fibrotic injury across multiple organ systems, including cardiovascular, pulmonary, and hepatic systems^18–20^. ARS expression levels, including those of EPRS1, are elevated in activated fibroblasts, and interestingly, in addition to its role in the translational pathway, EPRS1 has additional functions in nontranslational pathways, contributing to fibrotic scarring. Recently, studies on the role of EPRS1 in tubulointerstitial nephritis have reported that increased levels of EPRS1 in immune cells and fibrotic cells within the tissues of both mice and patients are associated with an increased composite kidney risk^21^. Therefore, EPRS1 appears to be an important factor in kidney diseases with fibrosis. However, there have been no reports on the role of EPRS1 in kidney fibrosis involving epithelial cells and fibroblasts.

In this study, we discovered that the expression levels of EPRS1 were elevated in the fibrotic kidneys of both patients and mice. Genetic *Eprs1* haploinsufficiency (*Eprs1*^+/−^) or pharmacological EPRS1 inhibition improved kidney fibrosis and dysfunction by inhibiting fibroblast activation and mitochondrial dysfunction. In vitro assays revealed that EPRS1 not only promoted proline synthesis but also stimulated ECM accumulation by activating fibroblasts, promoting the proliferation of fibroblasts, and impairing mitochondrial function in proximal tubules. Our findings collectively demonstrated that EPRS1 can mediate TGF-β-induced collagen accumulation to promote kidney fibrosis and dysfunction by activating fibroblasts and causing mitochondrial dysfunction.

## Materials and methods

### Patients and specimens

This study was approved by the Institutional Review Board (IRB) of Soonchunhyang University Cheonan Hospital (IRB number: 2021-10-014, Cheonan, Korea). The requirement to obtain informed consent from participants was exempted by the IRB. The experiments in this study were conducted following appropriate guidelines and regulations. A total of six patients were enrolled in this study, including three patients with focal segmental glomerulosclerosis and fibrosis confirmed by kidney biopsy. Nonfibrosis control specimens were obtained from the other three patients with minor glomerular changes (Supplementary Table 1). The expression levels of EPRS1 in kidney biopsy samples were evaluated using immunohistochemistry (IHC). EPRS1 expression was histopathologically assessed by a renal pathologist (J.H.L.) for all patients. Medical records were reviewed to analyze the correlations between EPRS1 and tubular atrophy, the fibrosis index, the estimated glomerular filtration rate (eGFR), and proteinuria. The fibrosis index was categorized into four scores according to pathology results: absent (scored as 0), mild (scored as 1), moderate (scored as 2), and severe (scored as 3). The eGFR was calculated according to the Chronic Kidney Disease Epidemiology Collaboration (CKD-EPI) equation^22^.

### Chemicals, siRNAs, and materials

The first-in-class EPRS1 inhibitor DWN12088 (Daewoong Pharmaceutical Co., Ltd., Yongin, Korea) was used in both in vitro and in vivo experiments^23,24^. Its chemical structure is shown in Supplementary Fig. 1. DWN12088 was stored at 10 mg/kg or 30 mg/kg in saline for in vivo experiments and at 1 mM in dimethyl sulfoxide (DMSO) for in vitro experiments. Small interfering RNA-mediated EPRS1 (siEPRS1) was purchased from Bioneer (Daejeon, Korea). The siEPRS1 sequences are provided in Supplementary Table 2. To knock down *EPRS1* gene expression, cells were treated with a mixture of two siRNAs (siEPRS1-1 and siEPRS1-2) targeting *EPRS1*. Recombinant TGF-β was purchased from R&D Systems (7754-BH). Water-soluble tetrazolium 1 (WST-1) assay solution was purchased from DoGenBio (Seoul, Korea). Tetramethylrhodamine methyl ester (TMRM) was obtained from Invitrogen (New York, NY, USA). Cycloheximide was purchased from Sigma‒Aldrich (St. Louis, MO, USA).

### Single-cell RNA sequencing (scRNA-seq)

All of the scRNA-seq methods used to analyze kidney tissues were described previously^25,26^. scRNA-seq was performed using whole kidneys from both the vehicle group (Veh) (*n* = 2) and the folic acid-induced kidney fibrosis (FA) mouse model (*n* = 2). scRNA-seq libraries were prepared via 10x chromium Next GEM Single-Cell 3’ Library and Gel Bead Kit v3.1 (10x Genomics, Pleasanton, CA, USA) and Chromium Single-Cell Chip G (10x Genomics) according to the manufacturer’s instructions. The cell suspensions were diluted in nuclease-free water to achieve a targeted cell count of 10,000. The cell suspension was mixed with master mix and loaded with single-cell 3’ v3.1 gel beads, and partitioning oil into chromium. Next, GEM Chip G. RNA transcripts from single cells were uniquely barcoded and reverse-transcribed within droplets. cDNA molecules were pooled. The cDNA pool then underwent an end repair process followed by the addition of a single ‘A’ base. It was then ligated with adapters. The purified libraries were quantified using quantitative polymerase chain reaction (qPCR) according to the qPCR quantification protocol guide (Kapa Biosystems, London, UK) and qualified using a 4200 Tape Station (Agilent Technologies, Santa Clara, CA, USA). Libraries were sequenced using the HiSeq platform (Illumina, San Diego, CA, USA) according to the read length in the user guide.

### Processing and analysis of scRNA-seq data

Cell Ranger (version 3.1.0, 10x Genomics) was used to process the scRNA-seq reads. To generate a gene expression matrix for each sample, we mapped the reads to the mm10 mouse transcriptome and produced a raw unique molecular identifier (UMI). Seurat v3.2.3 was used to analyze the gene expression matrix from each sample. Quality control filtering of cells and genes was performed using cells with at least 200 detected transcripts. Cells with more than 50 mitochondrial DNA read counts were excluded. To eliminate potential doublets, single cells with more than 8000 genes detected were filtered out. Finally, 19,297 single cells remained. They were applied in downstream analysis via Seurat following uniform manifold approximation and projection (UMAP) dimensional reduction. These single cells were clustered using the “FindNeighbors” (20 PCs for dimension reduction) and “FindClusters” functions (resolution = 2) in Seurat. First, total cells were classified into 39 distinct clusters. Similar clusters were merged on the basis of differentially expressed gene (DEG) numbers (10 < DEGs). To annotate cell identity to each cluster, we used conventional markers to categorize every cell into a known biological cell type. Next, the cells were classified into 16 clusters and annotated. The Seurat “Findallmarkers” function was used to identify preferentially expressed genes in clusters or DEGs between cell types. Pathway analyses were performed with gene set enrichment analysis (GSEA; https://www.gsea-msigdb.org/gsea/index.jsp). The results were visualized with a bar plot.

### Animal experiments

C57BL/6 EPRS1^tm1b^ heterozygous (*Eprs1*^+/−^) mice were obtained from the Toronto Center for Phenogenomics^27^. This mouse line was established by backcrossing Eprs1^tm1b+/−^; Cre- mice to C57BL/6NCrl followed by intercrossing tm1b^+/−^; Cre- progeny. *Eprs1*^+/+^ wild-type (WT) littermates were used as control mice (*Eprs1*^+/−^) in this study. Knockout alleles of *Eprs1* genes were routinely verified via qPCR analyses of ear tissues. All mouse procedures were performed following the Animal Research: Reporting of In Vivo Experiments (ARRIVE) 2.0 guidelines^28^. The mice were housed in a controlled environment with a 12-h/12-h light/dark cycle at a temperature of 25 °C. The experimental protocols were approved by the Institutional Animal Care and Use Committee of Soonchunhyang University (Asan, Korea) (SCH20-0038, SCH22-0039).

#### Experiment 1

To investigate the expression of EPRS1 in kidney fibrosis, 8-week-old male C57BL/6 mice (*n* = 7) were treated with a single dose of 250 mg/kg FA (dissolved in 0.3 M sodium bicarbonate) by intraperitoneal injection^29^. The FA mice presented characteristic injured tubules, interstitial fibrosis, and ECM accumulation^30–32^. Mice (*n* = 6) in the Veh group received 0.3 M sodium bicarbonate alone. The kidney function of the mice was confirmed to be significantly reduced at 28 days after FA injection.

#### Experiment 2

To investigate the effects of genetic inhibition of *Eprs1* on kidney fibrosis, the mice were divided into three groups: (1) *Eprs1*^+/+^ Veh mice (*n* = 4), (2) *Eprs1*^+/+^ FA mice (*n* = 6), and (3) *Eprs1*^+/−^ FA mice (*n* = 4). We administered FA to the mice once to induce kidney fibrosis. All of the mice were euthanized 28 days after FA injection, and kidney, urine, and blood samples were collected for further examination.

#### Experiment 3

To investigate the effects of pharmacological EPRS1 inhibition on kidney fibrosis, the mice were divided into five groups: (1) Veh + saline (*n* = 7), (2) Veh + 30 mg/kg EPRS1 inhibitor (*n* = 7), (3) FA + saline (*n* = 9), (4) FA + 10 mg/kg EPRS1 inhibitor (*n* = 9), and (5) FA + 30 mg/kg EPRS1 inhibitor (*n* = 9). An EPRS1 inhibitor (DWN12088)^24^ or vehicle was administered to the mice daily via intraperitoneal injection for 28 days. The mice were maintained in individual cages. All of the mice were euthanized, and kidney, urine, and blood samples were collected for further examination.

### Blood and urine analyses

Blood urea nitrogen (BUN) and serum creatinine levels in the mice were analyzed via a BUN kit (K024; Ann Arbor, MI, USA) and a creatinine kit (K625; BioVision, Milpitas, CA, USA), respectively. Urine samples were collected for 24 h and analyzed for protein and creatinine levels. Urine protein was quantified using a BCA assay (23225; Thermo Fisher Scientific, Waltham, MA, USA). Urinary creatinine was quantified using a kit (1012, Exocell, Philadelphia, PA, USA). The creatinine clearance of each mouse was calculated with the following equation based on a previous publication^33^: creatinine clearance (μl/min) = (urine creatinine concentration (mg/dl) * 24 h urine volume (μl))/(serum creatinine (mg/dl) * 24 * 60 min). All of the assay procedures were performed according to the manufacturer’s instructions.

### Cell culture and treatments

The rat kidney fibroblasts (NRK-49F, provided by Prof. Kim SW)^34^ and embryo fibroblasts (NIH3T3) were maintained in Dulbecco’s modified Eagle medium (DMEM) supplemented with 10% fetal bovine serum, 100 U/ml penicillin, and 100 g/ml streptomycin. Human proximal tubule (HK-2) cells were cultured in Dulbecco’s modified Eagle medium F12 (DMEM/F12) supplemented with 10% fetal bovine serum, 100 U/ml penicillin, and 100 g/ml streptomycin. NIH3T3 and HK-2 cells were purchased from the Korea Cell Line Bank (Seoul, Korea). The cells were incubated at 37 °C in a humidified atmosphere with 5% CO_2_. Experiments were conducted when the cells reached 80% confluence.

### Cell viability

The WST-1 assay was used to measure the viability of NRK-49F, NIH3T3, and HK-2 cells. Briefly, cells were seeded into 96-well plates and stabilized in culture media. The cells were then stimulated with the EPRS1 inhibitor at the indicated concentrations for 24 h to 48 h. WST-1/serum-free media solution (1:10) was added to the wells, and the plate was incubated at 37 °C for 2 h in a 5% CO_2_ humidified incubator. Finally, the absorbance was measured at 450 nm using a microplate reader (Multiskan Go; Thermo Fisher Scientific) at the Soonchunhyang Biomedical Research Core Facility of Korea Basic Science Institute (Cheonan, Korea).

### Western blotting

Total protein extraction and Western blot methods used to analyze kidney tissues and cells were described in previous studies^28,35^. Membrane or cytosol fractions were used according to the manufacturer’s protocol via the Mem-PER™ Plus Membrane Protein Extraction Reagent Kit (89842; Thermo Fisher Scientific). Immunoblot analysis was performed with the following antibodies: collagen Ι α1 chain (COL1A1, #72026; Cell Signaling Technology, Danvers, MA, USA), α-smooth muscle actin (α-SMA, #19245; Cell Signaling Technology), EPRS1 (NMS-01-0004; Curebio, Seoul, Korea), GAPDH (#5174; Cell Signaling Technology), β-actin (sc-47778; Santa Cruz, Dallas, TA, USA), fibronectin (FN, #63779; Cell Signaling Technology), TGF-β (#3711; Cell Signaling Technology), phospho-signal transducer and activator of transcription 3 (p-STAT3, #9145; Cell Signaling Technology), STAT3 (#9139; Cell Signaling Technology), phospho-SMAD family member 3 (p-Smad3, #9520; Cell Signaling Technology), Smad3 (#9523; Cell Signaling Technology), CTGF (ab6992, Abcam, Cambridge, UK), and horseradish peroxidase-conjugated secondary antibodies against rabbit IgG (#31466; Invitrogen, New York, NY, USA) and mouse IgG (W4021; Promega, Madison, WI, USA). Band intensities were quantitated via ImageJ software (National Institutes of Health; NIH, Bethesda, MD, USA).

### RNA isolation and qPCR

Total RNA was extracted from kidney tissues and cells using an AccuPrep Universal RNA Extraction Kit (K-3140; Bioneer, Daejeon, Korea) according to the manufacturer’s instructions. The RNA concentration was quantified based on the absorbance measured at 260 nm with a P200 spectrophotometer (Pultton, Ann Arbor, MI, USA). Total RNA was used for cDNA synthesis, followed by qPCR as described in a previous paper^35^. The sequences of the primer pairs used for qPCR are shown in Supplementary Table 3. The expression levels of the target genes were calculated using the 2^−^^ΔΔCt^ method^28^.

### Histological analysis

All procedures used to determine kidney pathology were described in a previous paper^28^. Briefly, kidney tissues were fixed in 10% neutral-buffered formalin, embedded in paraffin, sectioned to 3 μm in thickness, and stained with periodic acid-Schiff (PAS) or Masson’s trichrome (MT). IHC was carried out with a Novolink^TM^ polymer detection system (RE-7140-K; Leica Biosystems, Buffalo Grove, IL, USA) according to the manufacturer’s instructions. Deparaffinized samples were stained with COL1A1 (Cell Signaling Technology), α-SMA (Cell Signaling Technology), aquaporin-1 (AQP-1, ab168387; Abcam), EPRS1 (NMS-01-0004; Curebio), Ki67 (ab16667; Abcam), proliferating cell nuclear antigen (PCNA, #2586; Cell Signaling Technology), and p-STAT3 (Cell Signaling Technology) antibodies. Histological changes were confirmed under a microscope (DMi8; Leica Biosystems). For immunofluorescence (IF) staining, deparaffinized samples were stained for COL1A1 (Cell Signaling Technology), EPRS1 (NMS-01-0004; Curebio), lotus tetragonolobus lectin (LTL; FL-1321-2; Vector Laboratories, Newark, CA, USA), Ki67 (Abcam), vimentin (ab952547; Abcam), and α-SMA (F3777; Sigma Aldrich, St. Louis, MO, USA) antibodies. Alexa-488 (#A32731; Invitrogen) and Alexa-594 (#A32740; Invitrogen)-conjugated anti-rabbit IgG secondary antibodies were then used. Histological changes were confirmed via confocal microscopy (LSM710, Carl Zeiss, San Diego, CA, USA) at the Soonchunhyang Biomedical Research Core Facility of Korea Basic Science Institute (Cheonan, Korea). Quantitative analyses of all images were performed using ImageJ software.

### Mitochondrial morphology and functional analysis in vivo and in vitro

To check the mitochondrial structure, each sample was fixed in 3% glutaraldehyde for 24 h at 4 °C as previously described^36^. Images of each group of samples were taken via electron microscopy (EM) (HT7800; HITACHI, Chiyoda, Tokyo, Japan). ImageJ software was used to measure the mitochondrial length and aspect ratio (major axis/minor axis). The aspect ratio was determined as previously described^37^. To measure adenosine triphosphate (ATP) production both in vivo and in vitro, we used an ATP colorimetric assay kit (K354; BioVision). The ATP contents were normalized to the total protein concentration of each sample. To assess mitochondrial function in HK-2 cells, a TMRM kit (ab228569; Abcam) was used according to the manufacturer’s instructions. Hoechst 33342 (62249, Thermo Fisher Scientific) was added at a ratio of 1:2000, and the samples were incubated for 10 min for nuclear staining. Images were analyzed using confocal microscopy.

### Measurement of hydroxyproline content

To measure the collagen content in vivo, we used a hydroxyproline assay kit (STA-675; Cell Biolabs, San Diego, CA, USA) according to the manufacturer’s instructions. Frozen kidney tissues and cells were acid-hydrolyzed with 100 μl of 12 N HCl at 120 °C for 3 h. After evaporation, the supernatant was used for colorimetric assays.

### Measurement of protein translation levels

To demonstrate the effect of DWN12088 on protein translation levels in vitro, we used the Click-iT™ Plus O-propargyl-puromycin (OPP) Alexa Fluor™ 488 Protein Synthesis Assay Kit (C10456; Invitrogen), which measures ribosomal protein translation levels, following the manufacturer’s instructions. NRK-49F cells and HK-2 cells were incubated in DWN12088 or cycloheximide for 24 h. TGF-β stimulation was conducted with 5 ng/ml or 10 ng/ml TGF-β for 24 h. The cells were labeled with 10 μM Click-iT® OPP for 30 min and fixed with 3.7% formaldehyde for 15 min. Fluorescence was detected with confocal microscopy, and images were quantified with ImageJ software.

### Statistical analysis

All of the statistical analyses were performed using the GraphPad Prism 8.0 program (Dotmatics, MA, USA). All of the data are expressed as mean ± standard deviation. For the analysis of two groups, an unpaired two-tailed *t*-test was used. The results were also analyzed via one-way analysis of variance (ANOVA) for multiple comparisons of more than two groups, followed by Tukey’s post hoc test. Differences with *P-*values less than 0.05 were considered significant.

## Results

### EPRS1 expression levels are elevated in the fibrotic kidneys of humans and mice

**Fig. 1:**
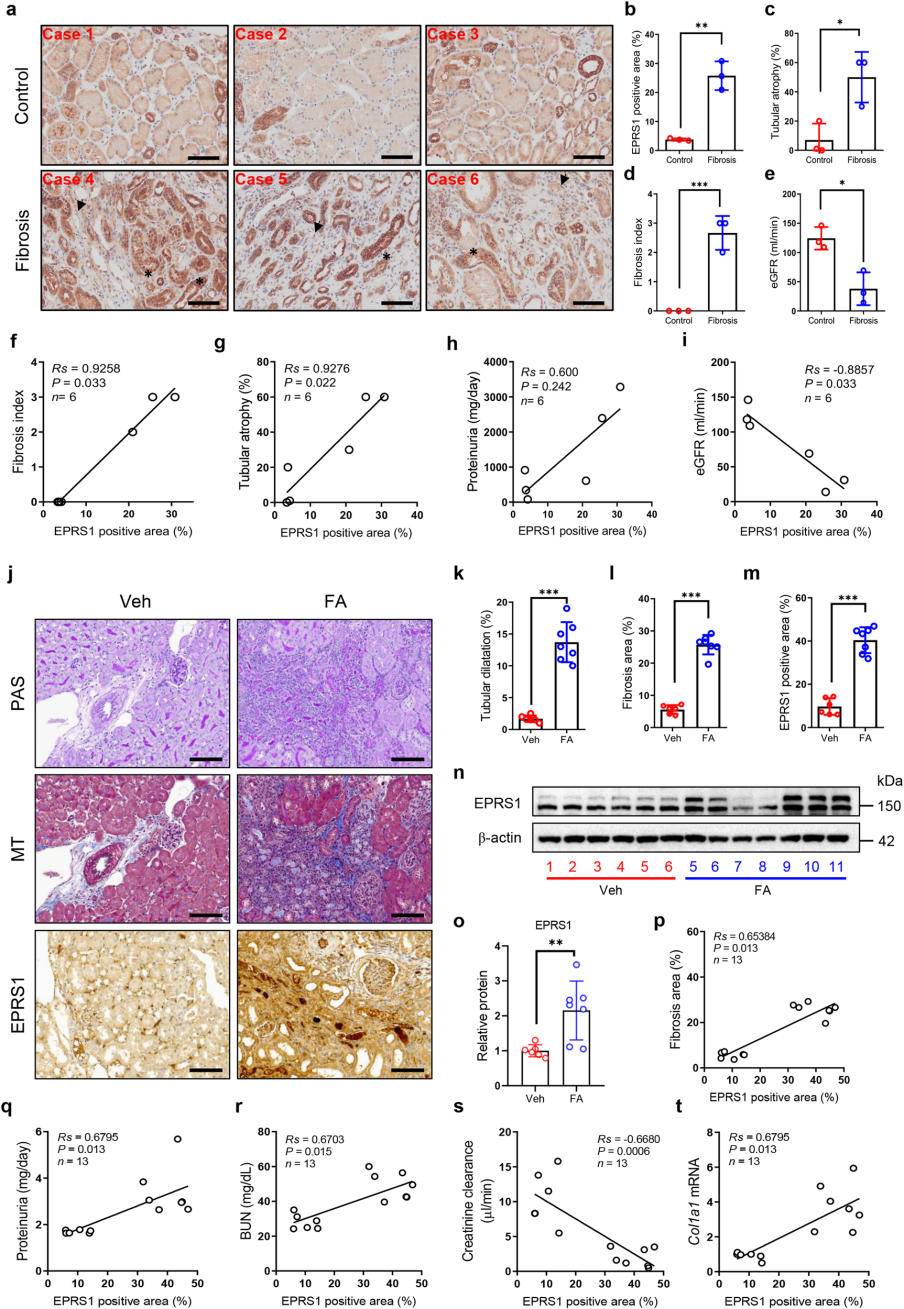
Glutamyl-prolyl-tRNA synthetase 1 (EPRS1) is upregulated in the fibrotic kidneys of humans and animals. **a** Representative image of immunohistochemistry (IHC) of EPRS1 in human kidneys. Normal control (Control) or fibrosis (Fibrosis) tissues were obtained from kidneys with minor glomerular changes or focal segmental glomerulosclerosis (FSGS) (*n* = 3 each). The arrowhead indicates fibroblasts. The asterisk indicates a proximal tubule cell. Scale bar = 100 μm. **b** The EPRS1-positive area was quantified via ImageJ analysis (*n* = 6). **c**–**e** Clinical indices of patients: tubular atrophy, fibrosis index, and estimated glomerular filtration rate (eGFR). **f**–**i** Linear regression revealed that the EPRS1-positive area was correlated with kidney dysfunction, as indicated by the fibrosis index, tubular atrophy, proteinuria, and eGFR. **j** Representative images of periodic acid-Schiff (PAS), Masson’s trichrome (MT), and IHC of EPRS1 in a folic acid-induced kidney fibrosis mouse model (FA mice). **k** Tubular dilatation as determined by measuring abnormal shapes in PAS-stained sections. **l**, **m** Fibrotic areas identified via MT staining and EPRS1-positive areas were quantified via ImageJ analysis (*n* = 6–7). Scale bar = 50 μm. **n**, **o** Representative Western blot and quantitative data of EPRS1 protein expression in kidneys from the three groups (*n* = 6–7). The numbers (1–11) indicate individual animals in a given group. **p**–**t** Linear regression revealing that the EPRS1-positive area correlated with kidney dysfunction: fibrosis area, proteinuria, blood urea nitrogen (BUN), creatinine clearance, and collagen Ι α1 (*Col1a1*) mRNA levels in FA-treated mice. The data are presented as mean ± standard deviation. Statistical data were analyzed by a two-tailed *t*-test. *P < 0.05, **P < 0.01, and ***P < 0.001. Veh vehicle.

We first determined whether EPRS1 levels were increased in pathologically fibrotic kidneys in humans. Compared with those in normal controls (Controls), the levels of EPRS1 expression were significantly increased in the kidneys of patients with focal segmental glomerulosclerosis (fibrosis, Fig. 1a, b) accompanied by atrophic tubules (Fig. 1a, c), interstitial fibrosis (Fig. 1a, d), and a decreased estimated glomerular filtration rate (eGFR, Fig. 1e). In addition, EPRS1 expression was positively correlated with the fibrosis index (Fig. 1f), tubular atrophy (Fig. 1g), and proteinuria (Fig. 1h) but negatively correlated with the eGFR (Fig. 1i).

We also examined whether the expression of EPRS1 was associated with kidney fibrosis in a mouse model of fibrotic damage to the kidneys. FA mice showed marked pathological changes (Fig. 1j–l) and kidney dysfunction accompanied by changes in BUN, serum creatinine, proteinuria, and creatinine clearance (Supplementary Fig. 2a–d). In addition, the mRNA levels of fibrosis markers (*Col1a1, Fn, Acta2*, and *Tgf-β*) and the *Eprs1* mRNA and protein levels were significantly elevated in the kidneys of FA mice compared with those in the Veh group (Fig. 1j, m–o; Supplementary Fig. 2e–i). The EPRS1-positive area was positively correlated with fibrosis, proteinuria, BUN levels, and *Col1a1* mRNA levels and negatively correlated with creatinine clearance (Fig. 1p–t). These results indicate that EPRS1 is associated with kidney fibrosis and dysfunction.

### Activated fibroblasts and injured proximal tubule cells are the main sources of EPRS1 in the fibrotic kidneys of humans and mice

**Fig. 2:**
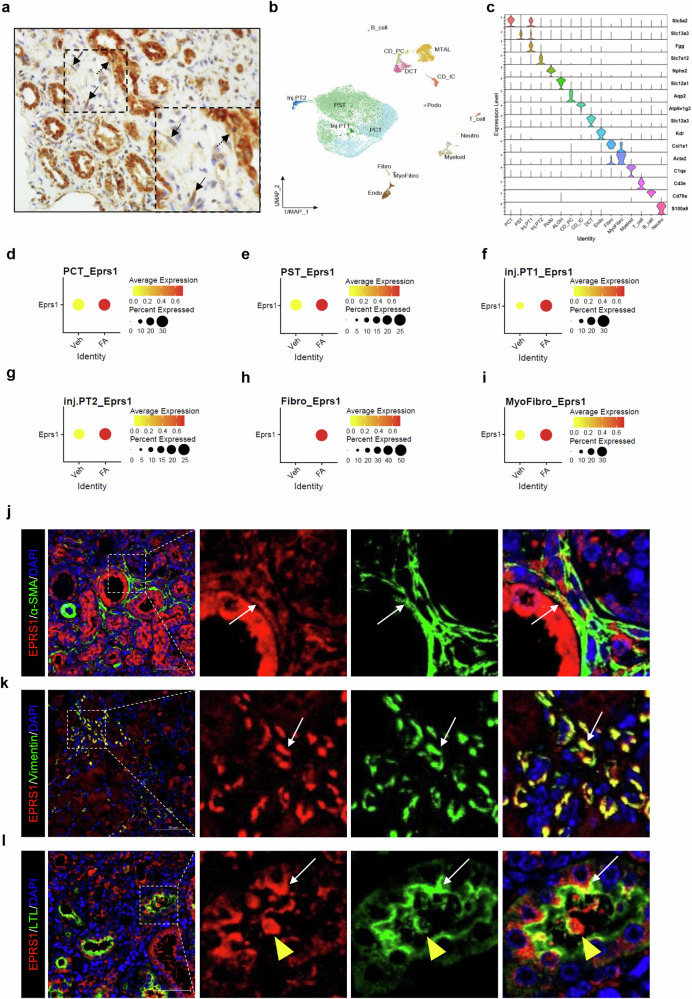
Single-cell RNA sequencing (scRNA-seq) profiling and immunostaining reveal the origins of EPRS1 in FA mice. **a** EPRS1-positive cell specificity showing an injured proximal tubule (dotted arrow) and fibroblasts (solid arrow) in human fibrotic tissue. **b** Uniform manifold approximation and projection (UMAP) plots showing total kidney cells expressed throughout the subcluster compared with those in FA mice and Veh mice. **c** Violin plot of specific marker genes that identified clusters generated via UMAP plotting. The color is different for various cell subtypes. The color of the cells represents the group’s origin. **d**–**i** Dot plot of *Eprs1* gene expression per cell cluster. **j**–**l** Representative confocal images of EPRS1 (red), α-smooth muscle actin (α-SMA, green), vimentin (green), and lotus tetragonolobus lectin (LTL, green) in FA mice. Scale bar = 50 μm. The colocalized portion is shown with a white arrow. The yellow arrowhead indicates a damaged proximal tubule in the lumen. PCT, proximal convoluted tubule; PST, proximal straight tubule; Inj. PT1, injured proximal tubule 1; Inj. PT2, injured proximal tubule 2; Podo, podocytes; ALOH, ascending limb of loop of Henle; CD_PC, principal cells of the collecting duct; CD_IC, intercalated cells of the collecting duct; DCT, distal convoluted tubule; Endo, endothelial cells; Fibro, fibroblast; MyoFibro, myofibroblast; Myeloid; T cell; B cell; Neutro, neutrophil.

In kidneys with fibrotic damage, EPRS1 was specifically localized in fibroblasts and injured proximal tubules (Fig. 2a). A total of 19,297 single cells were classified into 16 major cell types (Fig. 2b) on the basis of the expression of lineage-specific markers (Fig. 2c). We discovered that the gene expression level of *Eprs1* was dramatically enriched in specific cell types, such as proximal convoluted tubules (PCTs; *Slc5a2*), proximal straight tubules (PSTs; *Slc13a3*), injured proximal tubules 1 (Inj. PT1; *Slc5a2*, *Slc13a3*, and *Fgg*), injured proximal tubule 2 (Inj. PT2; *Slc7a12*), fibroblast (*Col1a1*), and myofibroblast (*Acta2*) of FA mice but not in podocytes (*Nphs2*), distal convoluted tubules (*Slc12a3*), intercalated cells of the collecting duct (*Atp6v1g3*), or principal cells (*Aqp2*). (Fig. 2d–i, Supplementary Fig. 3a–d). These findings were further validated by double IF staining, which revealed that EPRS1 colocalized with the markers α-SMA, vimentin, and LTL, which are markers of myofibroblasts, fibroblasts, and proximal tubules, respectively (Fig. 2j–l)^38,39^.

### EPRS1 promotes kidney dysfunction and fibrosis in FA mice

**Fig. 3:**
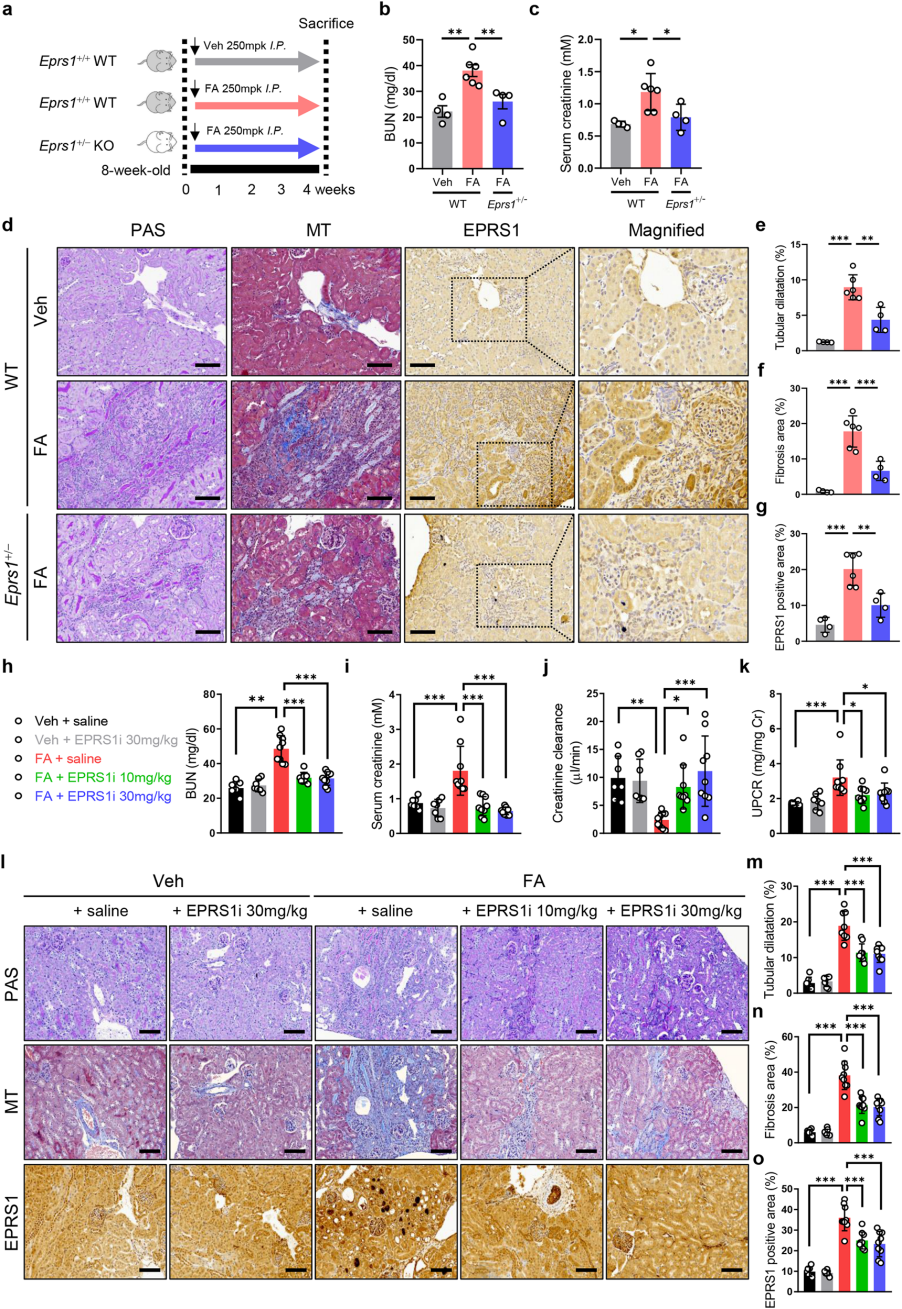
Genetic and pharmacological inhibition of EPRS1 attenuates kidney function and structure in FA mice. **a** Study designed to examine the effect of genetic *Eprs1* inhibition in FA mice. **b**, **c** BUN and serum creatinine levels in the three groups are indicated (*n* = 4–6). **d** Representative images of PAS, MT, and IHC staining of EPRS1 in kidney tissues (*n* = 4–6). **e**–**g** Tubular dilatation in the PAS-stained kidney, the fibrotic area in the MT-stained kidney, and the EPRS1-positive area were quantified via ImageJ analysis (*n* = 4‒6). Scale bar = 100 μm. **h**–**k** BUN levels, serum creatinine levels, creatinine clearance, and the urine protein-to-creatinine ratio (UPCR) in the five groups were measured to assess the effect of the EPRS1 inhibitor (*n* = 7–9). **l** Representative images of PAS, MT, and IHC staining of EPRS1 in kidney tissues (*n* = 7–9). **m**–**o** Tubular dilatation in the PAS-stained kidney, the fibrotic area in the MT-stained kidney, and the EPRS1-positive area were quantified via ImageJ analysis. The data are presented as mean ± standard deviation. Statistical data were analyzed by ANOVA with Tukey’s post hoc test. *P < 0.05, **P < 0.01, and ***P < 0.001.

FA mice with either genetic *Eprs1*^+/−^ or WT *Eprs1* were utilized to determine whether kidney function and structure were related to EPRS1 (Fig. 3a). Kidney dysfunction, indicated by increased BUN and serum creatinine levels, along with pathological findings such as the tubular dilatation and fibrosis observed in WT FA mice, were significantly reduced in *Eprs1*^+/−^ FA mice (Fig. 3b–f). IHC confirmed that the expression levels of EPRS1 were lower in *Eprs1*^+/−^ FA mice than in WT FA mice (Fig. 3d, g).

To further investigate the renoprotective effect of pharmacological EPRS1 inhibition in FA mice, we used the first-in-class EPRS1 inhibitor DWN12088. The mice were treated with this EPRS1 inhibitor through intraperitoneal injection at doses of 10 and 30 mg/kg for 28 days (Supplementary Fig. 4a). The results revealed that the EPRS1 inhibitor effectively protected against the detrimental effects of EPRS1 on kidney function, as indicated by the BUN level, serum creatinine level, creatinine clearance, and urine protein-to-creatinine ratio (UPCR) (Fig. 3h–k). Furthermore, the body weights (Supplementary Fig. 4b) and kidney weight/body weight ratios (Supplementary Fig. 4c) of FA mice treated with the EPRS1 inhibitor were significantly greater than those of FA mice not treated with the EPRS1 inhibitor. While examining the gross morphology of the kidneys, the small, bumpy, and constricted appearance of the kidneys of the FA mice was markedly improved after treatment with the EPRS1 inhibitor (Supplementary Fig. 4d). The decrease in the quantitative cross-sectional area of the kidneys after folic acid injection was also restored after EPRS1 inhibition (Supplementary Fig. 4e). Moreover, the EPRS1 inhibitor improved tubular dilatation and interstitial fibrosis and decreased EPRS1 protein levels, which was consistent with the findings in *Eprs1*^+/−^ FA mice (Fig. 3l–o). These data suggest that modulating EPRS1 by either genetic *Eprs1* haploinsufficiency or pharmacological EPRS1 inhibition can mitigate kidney dysfunction and fibrosis in FA mice.

### EPRS1 regulates collagen accumulation and fibrosis markers via proline synthesis in FA mice

**Fig. 4:**
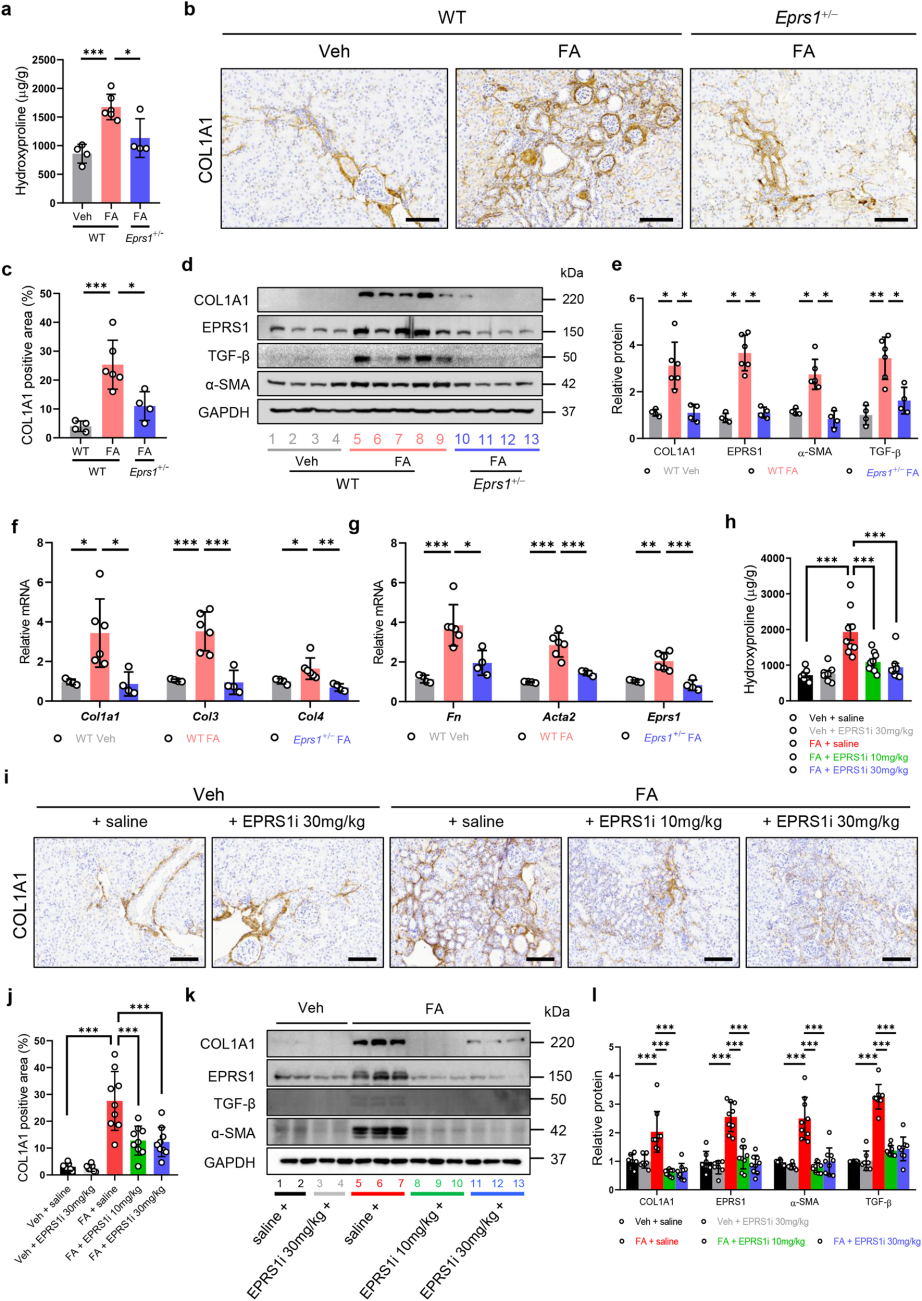
Genetic and pharmacological inhibition of EPRS1 reduces collagen accumulation and hydroxyproline levels. **a** Hydroxyproline was measured in whole kidney tissue from the three groups to examine the effect of genetic *Eprs1* inhibition in FA mice (*n* = 4–6). **b** Representative images of COL1A1 from IHC-stained kidneys. Scale bar = 100 μm. **c** The COL1A1-positive area was quantified by ImageJ analysis (*n* = 4–6). **d**, **e** Representative Western blot and quantitative data showing the protein expression levels of COL1A1, EPRS1, α-SMA, and transforming growth factor β (TGF-β) in kidneys from the three groups. The numbers (1–13) indicate individual animals in a given group. **f**, **g** The mRNA levels of *Col1a1, Col3, Col4, Fn, Acta2, and Eprs1* were analyzed via quantitative PCR (qPCR) and normalized to those of *Rpl13a* (*n* = 4–6). **h** Hydroxyproline was measured in whole kidney tissue from the five groups to assess the effect of the EPRS1 inhibitor (*n* = 7–9). **i** Representative images of COL1A1 from IHC-stained kidneys. Scale bar = 100 μm. **j** The COL1A1-positive area was quantified by ImageJ analysis (*n* = 7–9). **k**, **l** Representative Western blot and quantitative data of protein (COL1A1, EPRS1, α-SMA, and TGF-β) expression in kidneys from the five groups. The numbers (1–13) indicate individual animals in a given group. The data are presented as mean ± standard deviation. Statistical data were analyzed by ANOVA with Tukey’s post hoc test. *P < 0.05, **P < 0.01, and ***P < 0.001.

Given that EPRS1 synthesizes collagen^18^, we examined whether the regulation of EPRS1 could contribute to collagen accumulation. We found that the hydroxyproline content and COL1A1 accumulation were elevated in WT FA mice but decreased in *Eprs1*^+/−^ FA mice (Fig. 4a–c). In addition, compared with WT FA mice, *Eprs1*^+/−^ FA mice exhibited significantly lower protein levels of fibrosis markers (COL1A1, α-SMA, and TGF-β) and EPRS1 and lower mRNA expression levels of collagens (*Col1a1*, *Col3*, and *Col4*), *Fn*, *Acta2*, and *Eprs1* (Fig. 4d–g). FA mice treated with the EPRS1 inhibitor also presented reduced hydroxyproline content and COL1A1 accumulation, with decreased protein levels of COL1A1, α-SMA, TGF-β, and EPRS1 (Fig. 4h–l). Furthermore, while *Eprs1* and *Gars1* mRNA expression levels were increased in FA mice, catalytic EPRS1 inhibition significantly decreased *Eprs1* expression levels (Supplementary Fig. 4f, g). These data suggest that an EPRS1 inhibitor can protect against kidney fibrosis in FA mice.

### EPRS1 regulates fibroblast activation and proliferation in FA mice

**Fig. 5:**
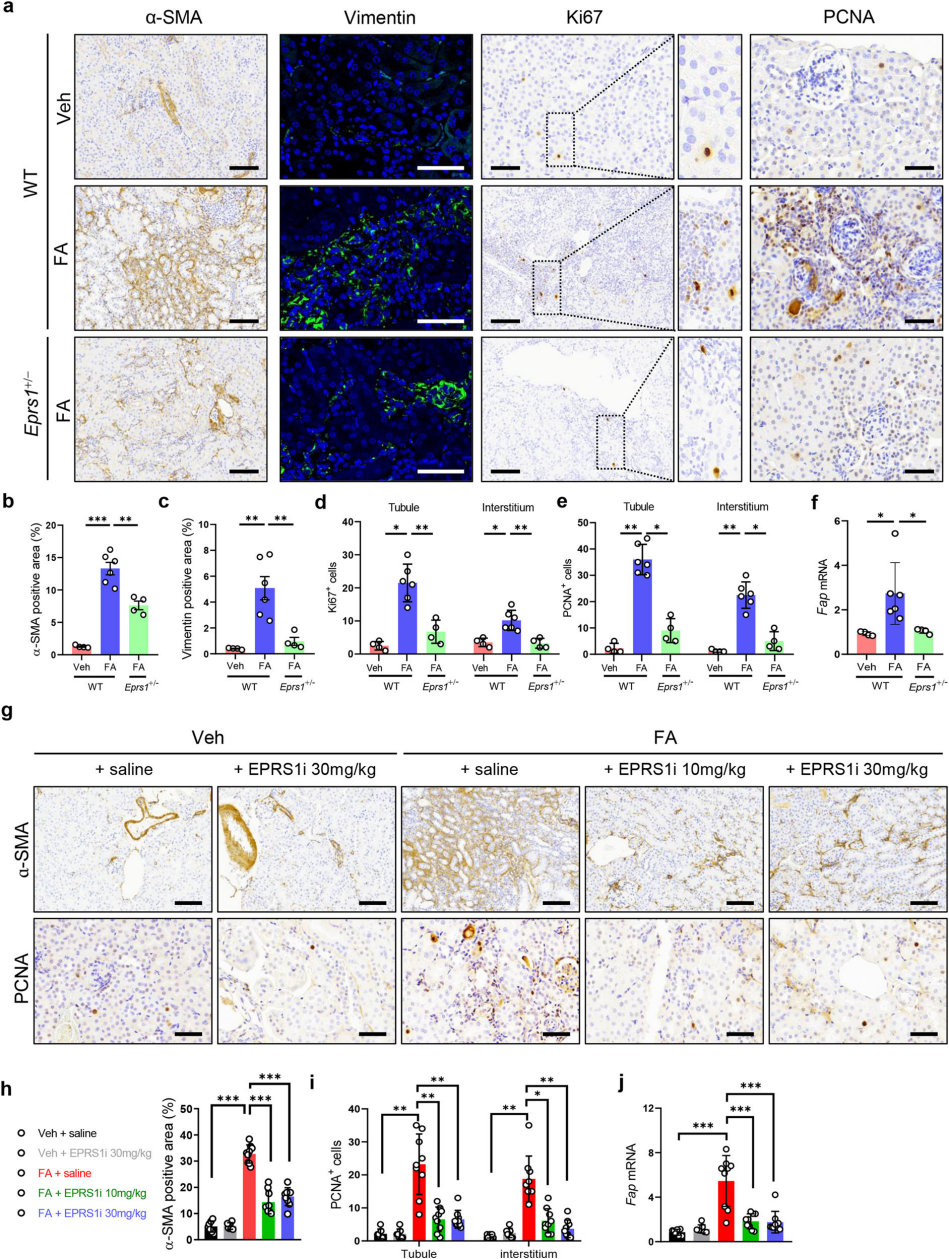
Genetic and pharmacological inhibition of EPRS1 suppresses fibroblast activation and proliferation in FA mice. **a** Representative images of α-SMA, vimentin, Ki67, and proliferating cell nuclear antigen (PCNA) from IHC-stained kidneys examining the effect of genetic *Eprs1* inhibition in FA mice. Scale bar = 100 μm (for α-SMA, vimentin, and Ki67), Scale bar = 50 μm (for PCNA). **b**–**e** α-SMA- and vimentin-positive areas and Ki67- and PCNA-positive cells were quantified by ImageJ analysis (*n* = 4‒6). **f** mRNA levels of fibroblast activation protein (*Fap*) were analyzed by qPCR and normalized to those of *Rpl13a* (*n* = 4–6). **g** Representative images of α-SMA and PCNA from IHC-stained kidneys used to assess the effect of the EPRS1 inhibitor. Scale bar = 100 μm (α-SMA), Scale bar = 50 μm (PCNA). **h** The α-SMA-positive area was quantified by ImageJ analysis (*n* = 7–9). **i** PCNA-positive cells were quantified by ImageJ analysis (*n* = 7–9). **j** mRNA levels of *Fap* were analyzed by qPCR and normalized to those of *Rpl13a* (*n* = 7–9). The data are presented as mean ± standard deviation. Statistical data were analyzed by ANOVA with Tukey’s post hoc test. *P < 0.05, **P < 0.01, and ***P < 0.001.

Since EPRS1 colocalized with markers of fibroblasts in FA mice (Fig. 2a), we stained for α-SMA, vimentin, PCNA, and Ki67 to determine whether EPRS1 affects fibroblast activation and proliferation in FA mice. The expression levels of the fibroblast activation markers α-SMA and vimentin were lower in *Eprs1*^+/−^ FA mice than in WT FA mice (Fig. 5a–c). Cell proliferation, as assessed by Ki67 and PCNA expression, was significantly lower in *Eprs1*^+/−^ FA mice than in WT FA mice, even though it was markedly increased in both the tubules and interstitium of WT FA mice (Fig. 5a, d, e). In addition, the mRNA levels of fibroblast activation protein (*Fap*) were increased in WT FA mice but decreased in *Eprs1*^+/−^ FA mice (Fig. 5f).

Consistently, treatment with an EPRS1 inhibitor suppressed fibrosis markers. Specifically, the EPRS1 inhibitor significantly reduced the expression levels of α-SMA and PCNA (Fig. 5g–i) and downregulated *Fap* mRNA levels (Fig. 5j) in FA-treated mice. These findings indicate that EPRS1 contributes to fibroblast activation and proliferation but that this effect is attenuated by the EPRS1 inhibitor.

### EPRS1 enhances the TGF-β pathway and modulates fibroblast activation in NRK-49F and NIH3T3 cells

**Fig. 6:**
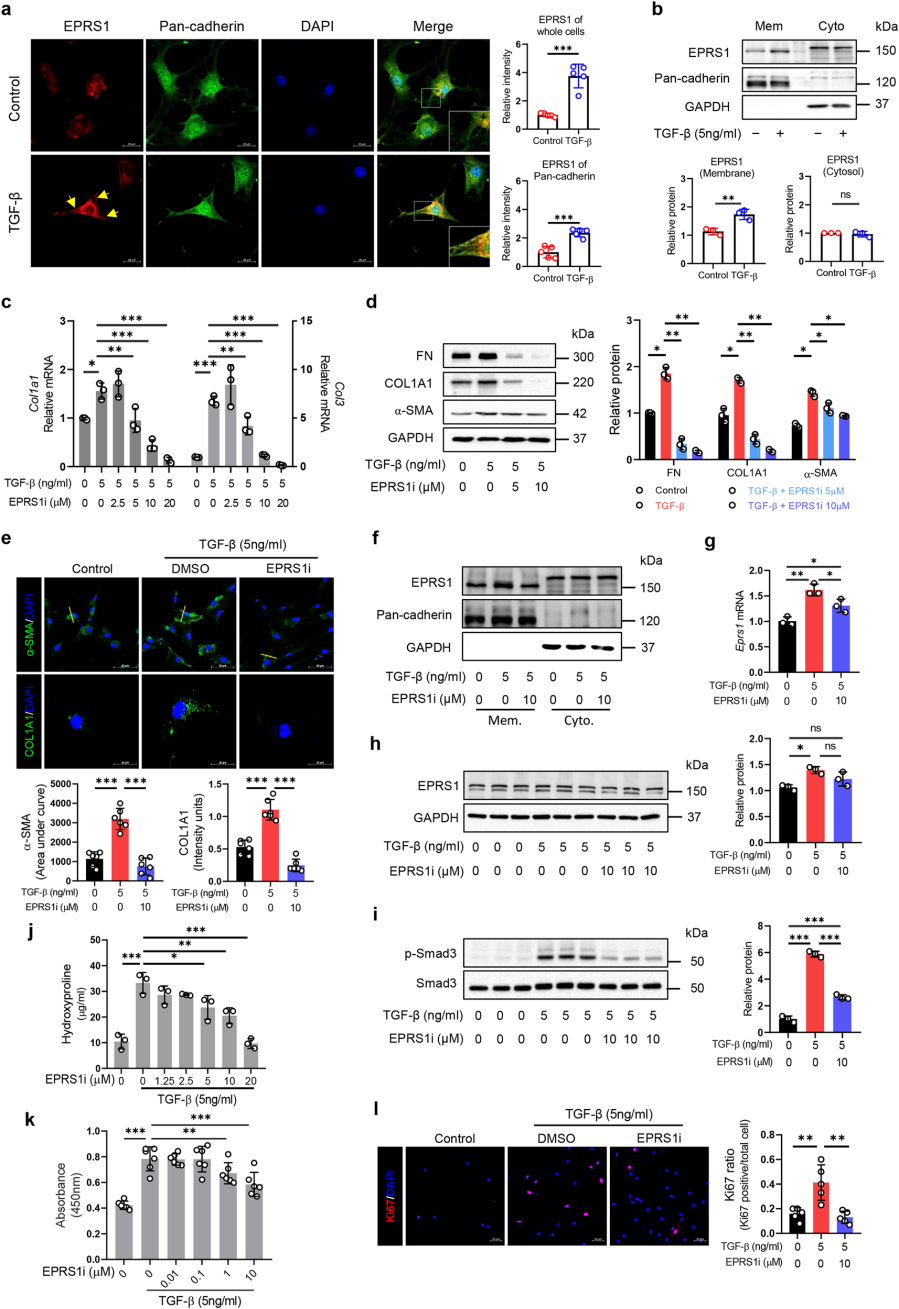
EPRS1 inhibitor suppresses fibroblast activation and proliferation in NRK-49F cells. **a** Representative confocal image (left) and quantitative data (right) of EPRS1 (red) and Pan-cadherin (green) in NRK-49F cells induced with TGF-β (5 ng/ml) for 24 h. The yellow arrow indicates the plasma membrane portion. Scale bar = 20 μm. Colocalization analysis was performed according to the methods provided in ZEN software. **b** Representative Western blot (top) and quantitative data (bottom) of the membrane and cytosol of NRK-49F cells treated with or without TGF-β (*n* = 3). Mem, membrane fraction; Cyto, cytosolic fraction. **c** Relative mRNA levels of *Col1a1* and *Col3* were analyzed via qPCR and normalized to *Gapdh* to assess the effect of the EPRS1 inhibitor (*n* = 3). **d** Representative Western blot (left) and quantitative data (right) of FN, COL1A1, and α-SMA in the whole lysate of EPRS1i-treated NRK-49F cells under TGF-β treatment conditions (*n* = 3). **e** Representative confocal images (top) and quantitative data (bottom) of COL1A1 and α-SMA in EPRS1i-treated cells under TGF-β treatment conditions for 24 h. The α-SMA-positive area and COL1A1-positive area were quantified by ImageJ analysis (*n* = 6). In particular, quantitative data for α-SMA were analyzed according to the area under the curve (AUC). The yellow line represents the value of the surface plot profile and the calculated area under the curve. **f** Membrane and cytosol of EPRS1i-treated NRK-49F cells incubated in the absence or presence of TGF-β for 24 h. **g** mRNA levels of *Eprs1* were analyzed by qPCR and normalized to *Gapdh* (*n* = 3). **h** Representative Western blot (left) and quantitative data (right) of EPRS1 expression in EPRS1i-treated cells under TGF-β treatment conditions for 24 h (*n* = 3). **i** Representative Western blot (left) and quantitative data (right) of protein (p-Smad3/Smad3) expression in EPRS1i-treated cells after 24 h of TGF-β treatment (*n* = 3). **j** The level of hydroxyproline in cultured cells was quantified via colorimetric assay (*n* = 3). **k** Proliferation of cells treated with the indicated concentrations of EPRS1i under TGF-β treatment conditions determined by a WST-1 assay. **l** Representative confocal images (left) of Ki67 in EPRS1i-treated cells under TGF-β treatment conditions for 24 h. The Ki67 ratio (right) was quantified by ImageJ analysis (*n* = 5). The data are presented as mean ± standard deviation. Statistical data were analyzed by ANOVA with Tukey’s post hoc test. *P < 0.05, **P < 0.01, and ***P < 0.001.

To determine how EPRS1 is involved in TGF-β-induced fibroblast activation in fibroblasts, NRK-49F cells were treated with TGF-β with or without the EPRS1 inhibitor. TGF-β treatment increased the expression of EPRS1, which is specifically translocated to the plasma membrane (Fig. 6a, b; Supplementary Fig. 5a). As expected, collagen mRNA levels and FN, COL1A1, and α-SMA protein levels were elevated in TGF-β-treated fibroblasts but significantly reduced by the EPRS1 inhibitor (Fig. 6c–e). Moreover, the translocation of EPRS1 was attenuated by its catalytic inhibition, with downregulated mRNA expression levels (Fig. 6f, g). The EPRS1 protein level tended to decrease, but not significantly (Fig. 6h). Cytotoxicity evaluations indicated that treatment with the EPRS1 inhibitor at concentrations less than 20 μM was not cytotoxic (Supplementary Fig. 5b). In addition, the impact on the global translation level of the EPRS1 inhibitor was minimal before TGF-β treatment in NRK-49F cells (Supplementary Fig. 5c, d). In fibroblasts activated by TGF-β, the EPRS1 inhibitor significantly decreased the global translation level, even though the translation level was significantly greater than that in inactivated fibroblasts (Supplementary Fig. 5e, f).

To identify the signaling pathway that enhances fibroblast activation in fibroblasts, we identified the pathway with a normalized enrichment score in our single-cell data. The pathway associated with increased activity in the fibroblasts of FA mice was closely associated with the TGF-β pathway (Supplementary Fig. 6). Therefore, the TGF-β-Smad pathway was analyzed and validated in vitro, which confirmed that EPRS1 inhibition reduced the increase in p-Smad3 induced by TGF-β (Fig. 6i). Furthermore, the EPRS1 inhibitor decreased the TGF-β-induced increase in hydroxyproline levels starting at 5 μM (Fig. 6j). Additionally, the EPRS1 inhibitor significantly reduced TGF-β-induced cell proliferation, as demonstrated by a WST-1 assay (Fig. 6k) and Ki67 staining (Fig. 6l).

Moreover, the effects of the EPRS1 inhibitor were further validated in another fibroblast cell line, NIH3T3. EPRS1 was observed to be located on the plasma membrane and in the cytosol under TGF-β treatment conditions (Supplementary Fig. 7a). Since cell viability after the EPRS1 inhibitor treatment was significantly decreased at a concentration of 2.5 μM (Supplementary Fig. 7b), we treated cells with the EPRS1 inhibitor at a concentration of 1 μM. The Western blot and IF staining results were consistent with those of NRK-49F, which exhibited decreased COL1A1 and α-SMA expression through the Smad3 pathway and decreased expression levels of EPRS1, α-SMA, and collagen and decreased cell proliferation after EPRS1 inhibitor treatment (Supplementary Fig. 7c–l). These results demonstrate that the EPRS1 inhibitor has antifibrotic effects on both NRK-49F and NIH3T3 cells, indicating that EPRS1 plays a critical role in fibroblast activation via the TGF-β pathway.

### EPRS1 regulates p-STAT3 expression and fibrosis markers in the proximal tubule

The role of EPRS1 in the proximal tubules in TGF-β-induced fibrotic injury was investigated in HK-2 cells. Under TGF-β treatment conditions, the translocation of EPRS1 to the cell membrane occurred, and the levels of *EPRS1* and fibrosis markers (*COL1A1*, *ACTA2*, and *FN)* were elevated but attenuated by *EPRS1* knockdown (Supplementary Fig. 8a–k). Pharmacological inhibition of EPRS1 consistently decreased the increase in the expression of fibrotic proteins and hydroxyproline induced by TGF-β, with cell viability maintained at concentrations less than 10 μM and minimal effects on global translation levels (Supplementary Figs. 9a–e, 10a–h). Furthermore, the increase in the mRNA and protein levels of profibrotic factors such as *TGF-β* and *CTGF* in response to TGF-β stimulation was reduced in EPRS1 inhibitor-treated cells (Supplementary Fig. 10i, j).

**Fig. 7:**
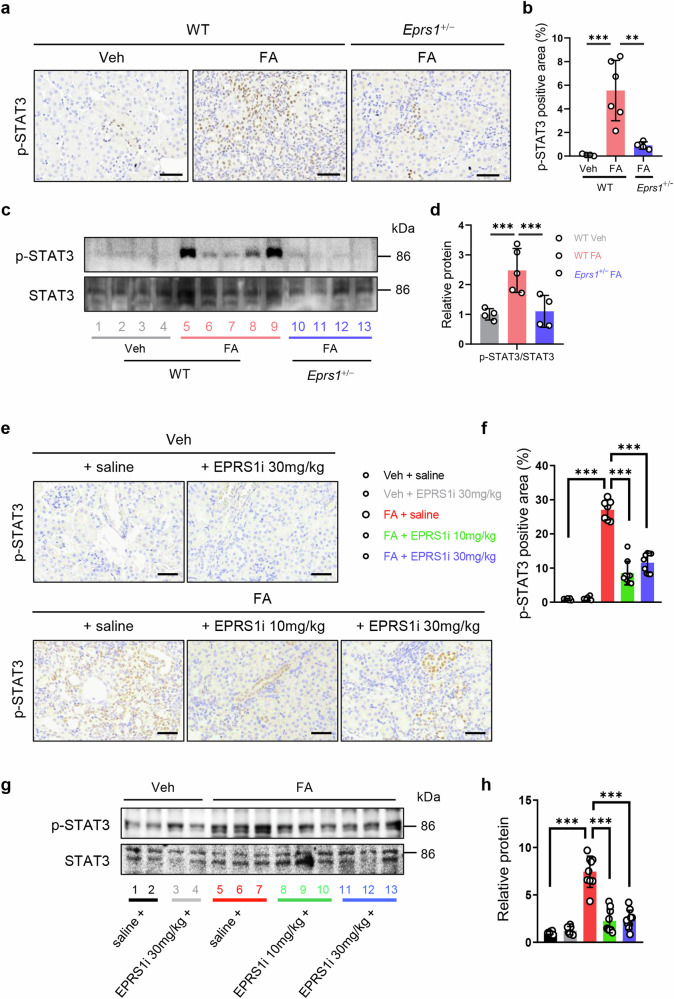
Genetic and pharmacological inhibition of EPRS1 decreases p-STAT3 in FA mice. **a** Representative images of phospho-signal transducer and activator of transcription 3 (p-STAT3, brown) from an IHC-stained kidney showing the effect of genetic *Eprs1* inhibition in FA mice. Scale bar = 50 μm. **b** The p-STAT3-positive area was quantified by ImageJ analysis (*n* = 4–6). **c**, **d** Representative Western blot and quantitative data of protein (p-STAT3/STAT3) expression in kidneys from the three groups. The numbers (1–13) indicate individual animals in a given group (*n* = 4–6). **e**, **f** Representative images and quantitative data showing the expression of p-STAT3 (brown) along with nuclei (blue) in each group to assess the effect of the EPRS1 inhibitor (*n* = 7–9). Scale bar = 50 μm. **g**, **h** Representative Western blot and quantitative data of protein (p-STAT3/STAT3) expression in the kidneys of the five groups. The numbers (1–13) indicate individual animals in a given group (*n* = 7–9). The data are presented as the means ± standard deviations. Statistical data were analyzed by ANOVA with Tukey’s post hoc test. *P < 0.05, **P < 0.01, and ***P < 0.001.

We conducted a functional annotation analysis of the DEGs in the scRNA-seq data to further identify the noncanonical pathway of EPRS1 in injured PT cells, specifically in Inj. PT1 and PT (PCT + PST). The expression of genes increased in *Eprs1*-positive Inj. PT1 cells were more significantly associated with Janus kinase signal transducer and activator of transcription (JAK-STAT) signaling than with the TGF-β signaling pathway (Supplementary Fig. 11a). GSEA revealed that the *Stat3* gene was upregulated in Inj. PT1 relative to *Eprs1*-positive PT cells (Supplementary Fig. 11b, c). These results were further confirmed in *Eprs1*^+/−^ FA mice and FA mice treated with the EPRS1 inhibitor (Fig. 7a–h), HK-2 cells with *EPRS1* knockdown (Supplementary Fig. 11d, e), and pharmacologically inhibited EPRS1 (Supplementary Fig. 11f, g), which presented decreased p-STAT3 expression. These results suggest that the antifibrotic effect of EPRS1 is associated with its regulatory role in the STAT3 pathway.

### EPRS1 regulates mitochondrial damage in the proximal tubule

**Fig. 8:**
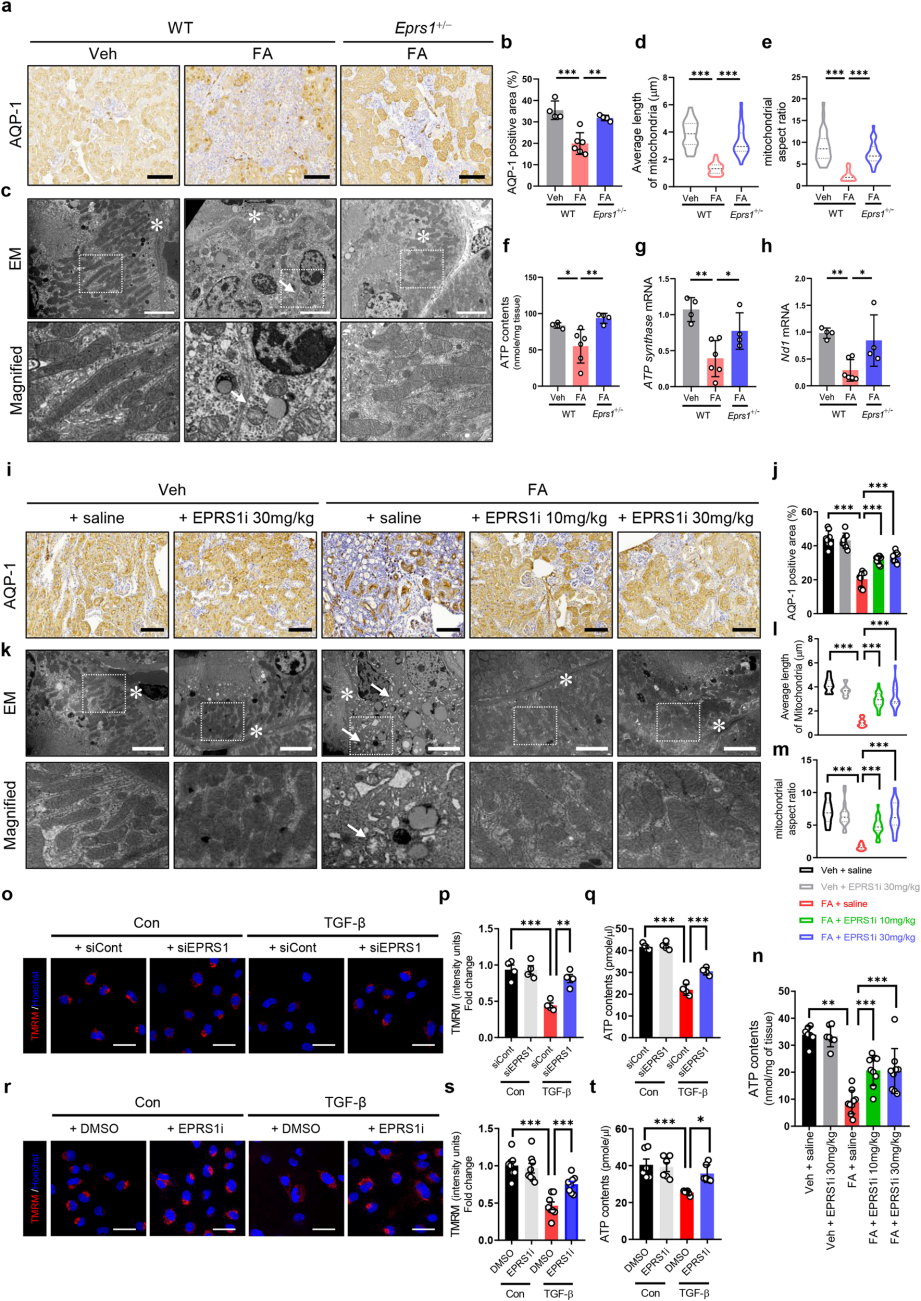
Genetic and pharmacological inhibition of EPRS1 improves mitochondrial dysfunction in FA mice and HK-2 cells. **a** Representative images of aquaporin-1 (AQP-1) from IHC-stained kidneys. Scale bar = 100 μm. **b** The AQP-1-positive area was quantified by ImageJ analysis (*n* = 4–6). **c** Representative electron microscopy (EM) micrographs showing the effects of genetic *Eprs1* inhibition on FA mice. The asterisk indicates tubular basement membrane thickening. The arrows indicate swollen mitochondria in each group (*n* = 4–6). Scale bar = 5 μm. **d**, **e** Quantitative data on the mitochondrial length and aspect ratio (ratio of large to small axes) were analyzed by ImageJ (*n* = 4–6). **f** Adenosine triphosphate (ATP) contents in whole kidney tissues from the three groups (*n* = 4–6). **g**, **h** Relative mRNA levels of *ATP synthase* and *NADH dehydrogenase 1 (ND1)* were analyzed via qPCR and normalized to those of *18S rRNA* (*n* = 4–6). **i** Representative images of AQP-1 from IHC-stained kidneys used to assess the effect of the EPRS1 inhibitor. Scale bar = 100 μm. **j** The AQP-1-positive area was quantified by ImageJ analysis (*n* = 7–9). **k** Representative EM micrographs of kidney fibrosis in FA mice with or without EPRS1i treatment. The asterisk indicates tubular basement membrane thickening. The arrow indicates swollen mitochondria in FA mice. Scale bar = 5 μm. **l**, **m** Quantitative data on the mitochondrial length and aspect ratio were analyzed using ImageJ (*n* = 7–9). **n** ATP contents in whole kidney tissues from the five groups (*n* = 7–9). **o**, **p** Representative confocal images and quantitative data showing tetramethylrhodamine methyl ester (TMRM) activity in siEPRS1 (20 nM)-treated HK-2 cells under TGF-β (10 ng/ml) treatment conditions for 24 h (*n* = 4). Scale bar = 50 μm. **q** ATP contents in siEPRS1 (20 nM)-treated cells under TGF-β (10 ng/ml) treatment conditions were determined according to the manufacturer’s protocol (*n* = 4). Scale bar = 50 μm. **r**, **s** Representative confocal images and quantitative data showing TMRM in EPRS1i (1 μM)-treated cells under TGF-β (10 ng/ml) treatment conditions for 24 h (*n* = 8). Scale bar = 50 μm. **t** ATP contents in EPRS1i (1 μM)-treated cells under TGF-β (10 ng/ml) treatment conditions (*n* = 6). The data are presented as mean ± standard deviation. Statistical data were analyzed by ANOVA with Tukey’s post hoc test. *P < 0.05, **P < 0.01, and ***P < 0.001. siEPRS1: siRNA-mediated EPRS1.

An in vivo study revealed that AQP-1, a PT marker^40^ which was decreased in WT FA mice, was significantly improved in *Eprs1*^+/−^ FA mice (Fig. 8a, b). Additionally, the impact of EPRS1 on mitochondrial dysfunction was investigated via scRNA-seq data, and changes in apoptosis, the citrate cycle, and fatty acid metabolism pathways were observed in *Eprs1*-positive PT cells (Inj. PT1 *vs*. PCT + PST, Supplementary Fig. 12a). Tubules in WT FA mice exhibited a thickened tubular basement membrane, injured mitochondria with swollen and deficient cristae and lower mitochondrial length and aspect ratios in EM images (Fig. 8c–e), with decreased ATP contents (Fig. 8f) and mRNA levels of *ATP synthase* and *NADH dehydrogenase 1* (*Nd1*) (Fig. 8g, h)^41,42^. However, they were markedly improved in *Eprs1*^+/−^ FA mice. The AQP-1-positive area (Fig. 8i, j), mitochondrial morphology and function (Fig. 8k–n), and the expression of mitochondrial marker genes such as *ATP synthase* (Supplementary Fig. 12b) and *Nd1* (Supplementary Fig. 12c) were restored in FA mice treated with the EPRS1 inhibitor.

We further examined the impact of genetic *EPRS1* inhibition by siRNA on mitochondrial dysfunction via the mitochondrial membrane potential marker TMRM and ATP content in vitro. TGF-β treatment decreased the TMRM signal (Fig. 8o, p) and ATP content (Fig. 8q), which were restored after *EPRS1* knockdown. Similarly, mitochondrial dysfunction was restored by pharmacological inhibition of EPRS1 (Fig. 8r–t). In summary, EPRS1 can induce mitochondrial dysfunction and tubular injury, which are alleviated after EPRS1 inhibition.

## Discussion

**Fig. 9:**
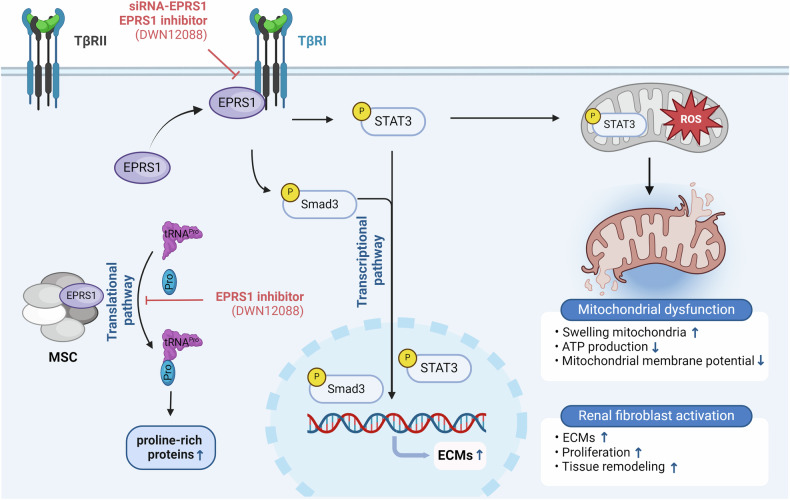
Schematic showing that EPRS1-mediated fibroblast activation and mitochondrial dysfunction induce kidney fibrosis. Our results indicate that EPRS1 can mediate kidney fibrosis. EPRS1 is an enzyme that catalyzes the binding of proline to tRNA^Pro^. EPRS1 of the multi-tRNA synthetase complex (MSC) translationally synthesizes proline-rich profibrotic proteins. However, the EPRS1 inhibitor inhibits hydroxyproline. EPRS1 also activates the TGF-β pathway. EPRS1 can translocate to the cell membrane and bind to TGF-β receptor 1 (TβRI), resulting in downstream pathways. Our results demonstrated that EPRS1 is linked to three different pathways associated with kidney fibrosis. EPRS1 regulates the phosphorylation of STAT3 and Smad3. The nuclear localization of p-STAT3 and p-Smad3 induces the transcription of profibrotic genes. This pathway leads to fibroblast activation, resulting in increased ECM production, cell proliferation, and tissue remodeling. p-STAT3 affects mitochondrial reactive oxygen species (ROS) generation^45,46^. Eventually, ROS lead to mitochondrial dysfunction in cells. However, EPRS1 inhibition not only transcriptionally reduces p-Smad3 and p-STAT3 but also improves mitochondrial dysfunction. As a result, EPRS1 inhibition reduces kidney fibrosis through transcriptional and translational regulation. MSC multi-tRNA synthetase complex, ECM extracellular matrix, EMT epithelial‒mesenchymal transition, FMT fibroblast–mesenchymal transition, Pro proline, ROS reactive oxygen species. This figure was created with Biorender.com.

The results of the present study demonstrated that EPRS1 is a key driver of kidney fibrosis and dysfunction. First, EPRS1 was upregulated in the fibrotic kidneys of patients and mice, particularly in fibroblasts, myofibroblasts, and proximal tubular cells (PCT, PST, Inj. PT1, and Inj. PT2). Second, EPRS1 mediated the accumulation of hydroxyproline and ECM proteins in kidney fibrotic injury through canonical pathways. Third, EPRS1 controlled fibroblast activation both in vivo and in vitro via the Smad3 pathway in fibroblasts. Fourth, EPRS1 was associated with the JAK-STAT pathway and mitochondrial dysfunction in the proximal tubule. This finding implies that Smad3 and STAT3 together might contribute to further kidney damage, as suggested by previous studies^41,42^. Furthermore, EPRS1 inhibition decreased both Smad3 and STAT3 in the kidney. These findings indicate that EPRS1 contributes to kidney fibrosis and dysfunction through canonical and noncanonical signaling pathways in fibroblasts and PT cells (Fig. 9).

The canonical function of EPRS1 in the multi-tRNA synthetase complex (MSC) is to link proline to tRNA^Pro^, an important process for synthesizing proline-rich proteins such as collagens^14^. We confirmed that targeting EPRS1 could attenuate kidney fibrogenesis by decreasing the production of proline-rich proteins in fibroblasts and proximal tubules. On the other hand, its noncanonical function involves the regulation of the TGF-β pathway, particularly through its interaction with TGF-β receptor I (TβR1)^43^. Our study confirmed that TGF-β stimulation promotes increased EPRS1 expression, with its translocation to cell membranes in fibroblasts and epithelial cells. Previous studies revealed that the levels of ARSs increase in TGF-β-treated fibroblasts, which is considered a mechanism that supports protein synthesis^18,20^. The interaction between translocated EPRS1 and TβR1 increased Smad2/3 phosphorylation, activating the TGF-β pathway. An additional noncanonical function of EPRS1 is to activate the JAK-STAT pathway in proximal tubule cells. In particular, in Inj. PT1, which have high EPRS1 expression, we observed an increase in the expression of STAT3. p-STAT3 is known to transcriptionally regulate the generation of fibrotic factors^44^ and enter the mitochondria, inducing the production of reactive oxygen species^45,46^. Our study revealed that inhibiting EPRS1 decreases the phosphorylation of STAT3 and fibrotic factors and improves mitochondrial dysfunction and morphology under fibrotic conditions.

Mitochondrial dysfunction is recognized as a key contributor to the pathogenesis of CKD^9,47^. The kidneys, particularly tubular cells, are highly dependent on mitochondrial function, second only to the heart. In kidney fibrosis, impaired mitophagy and alterations in mitochondrial enzymes and metabolic pathways contribute to the exacerbation of tissue injury. Therefore, our findings suggest that targeting EPRS1 with novel therapeutic agents may be a promising strategy for mitigating mitochondrial dysfunction and improving renal outcomes in CKD patients.

The role of EPRS1 in injury and fibrosis has been extensively studied across various organ systems, including the heart, liver, lung, and immune system^18–20,24,27,43,48^. In cardiac fibrosis, the levels of ARSs, particularly EPRS1, are increased in TGF-β-treated human cardiac fibroblasts, with a significant correlation between EPRS1 levels and collagen levels in human heart tissue^18^. Additionally, *Eprs1* haploinsufficiency was shown to reduce heart hypertrophy. In liver stellate cells, phosphorylated EPRS1, in response to TGF-β stimulation, is bound to TβR1 and facilitates the phosphorylation of SMAD2/3, contributing to fibrosis^20,43^. This finding aligns with our findings in kidney fibroblasts, where EPRS1-stimulated TβR1 activated the Smad3 pathway, leading to collagen and α-SMA synthesis. Furthermore, EPRS1 induced fibrosis via the JAK-STAT pathway in type 2 alveolar epithelial cells, a mechanism consistent with our findings in kidney epithelial cells^19^. In tubulointerstitial nephritis, *Eprs1* expression is increased in NK cells and γδ T cells in the early phase, and these cells are involved in the immune response^21^. Furthermore, *Eprs1*^+/−^ mice exhibited relatively improved mitochondrial structure and function, similar to our findings.

We confirmed that the first-in-class EPRS1 inhibitor, DWN12088, effectively reduced kidney fibrosis and dysfunction through both canonical and noncanonical pathways without adverse effects. DWN12088 selectively inhibited proline-rich proteins with minimal effects on other proteins, which is consistent with previous research^24^. This specificity is likely due to the slower translation process of proline compared to other amino acids, making peptides with proline‒proline motifs more sensitive to mild EPRS1 inhibition^18,49,50^. Consequently, EPRS1 may be a critical therapeutic target for fibrosis attenuation^21^.

DWN12088-treated mice showed significant recovery of tubular dilatation and fibrosis areas, and their kidney function indicators, such as BUN and creatinine levels, returned to normal ranges. This normalization of BUN and creatinine levels, despite incomplete structural recovery, can be attributed to the functional compensatory mechanisms of the kidneys^51^. In the context of kidney impairment, the remaining kidney parenchyma can increase its filtration capacity and waste removal through compensatory mechanisms, leading to increased functionality of the surviving glomeruli. This adaptive response maintains the overall kidney filtration capacity assessed by kidney function indicators such as BUN and serum creatinine levels within normal physiological limits, despite incomplete structural recovery, such as tubular dilatation and fibrosis areas. In our study, mice with severely damaged kidneys, which were unable to compensate, exhibited functional recovery after EPRS1 inhibitor treatment, although incomplete structural restoration was observed.

DWN12088 has significantly lower toxicity than halofuginone, a previously discovered EPRS1 inhibitor, with fewer side effects, such as organ failure and gastrointestinal toxicity^48,52^. Interestingly, DWN12088 treatment reduced *Eprs1* mRNA expression, despite being a catalytic inhibitor. These findings contrast with those of halofuginone treatments in hepatic stellate cells, which exhibited no decrease in *EPRS1* expression levels even though collagen synthesis and α-SMA expression were reduced^20^.

The observed discrepancies in the mechanisms of action between halofuginone and DWN12088, both recognized as EPRS1 inhibitors, likely arise from differences in their interactions with EPRS1^24^. Although both compounds act as catalytic inhibitors of EPRS1, their binding structures to EPRS1 are different. Specifically, halofuginone binds symmetrically to the catalytic site of the protomer of the EPRS1 dimer, whereas DWN12088 exhibits asymmetric binding with varying affinities. Additionally, while ATP is crucial for EPRS1 enzyme function and is facilitated through hydrogen bonding at the catalytic site, the administration of DWN12088 resulted in the loss of ATP binding by protomer B of EPRS1.

During the fibroblast-to-myofibroblast transition across various organ systems, the upregulation of *EPRS1* expression has been observed after TGF-β stimulation^18,20^. It is hypothesized that an as-yet-uncharacterized mechanism induces EPRS1 expression during this transition. DWN12088 inhibited this upregulation, whereas halofuginone did not have the same effect. Our results, which are consistent both in vivo and in vitro, suggest that there may be an undiscovered mechanism at play, rather than these findings being attributable to chance. This interpretation necessitates further validation through subsequent studies.

Our study has several limitations. We did not determine the inhibitory effect of EPRS1 on TβR1. Although phosphorylated EPRS1 is known to influence the phosphorylation of Smad3 and STAT3^19,43^, we have not confirmed this in our current research. In addition, even though the toxicity of DWN12088 has been evaluated in normal mice in previous studies^24^, drug toxicity in FA mice was not assessed in this study. Moreover, even though other ARSs in addition to EPRS1, such as GARS1, had increased expression levels during fibrosis, their role in kidney fibrosis was not investigated in this study. However, our results demonstrated for the first time that EPRS1 is increased in fibroblasts and injured proximal tubules in CKD, confirming that DWN12088 has potential as a novel agent for CKD treatment.

## Supplementary information

Supplementary information
