# Supplementary material for: EPRS1-mediated fibroblast activation and mitochondrial dysfunction promote kidney fibrosis
Source: Exp Mol Med. 2024 Dec 2;56(12):2673–89. doi: 10.1038/s12276-024-01360-6 (PMC11671583; doi:10.1038/s12276-024-01360-6)
Supplement: Supplementary file 1 — Supplementary information [file 12276_2024_1360_MOESM1_ESM.pdf]

Supplementary Table 1. Baseline clinical characteristics of patients

Case	Sex	Age (years)	BP (mmHg)	Serum creatinine (mg/dl)	Proteinuria (mg/day)	eGFR (ml/min)	Tubular atrophy (%)	Pathologic diagnosis	Fibrosis index
1	M	18	110/60	1.0	78	109	0	Minor glomerular change	0
2	F	21	100/60	0.7	913	118	20	Minor glomerular change	0
3	M	24	120/80	0.5	345	146	1	Minor glomerular change	0
4	M	62	120/70	1.1	3284	69	30	FSGS	2
5	F	44	180/100	3.5	2394	14	60	FSGS	3
6	F	51	130/80	1.8	613	31	60	FSGS	3

The fibrosis index was categorized into four scores according to pathology results: absent (scored as 0), mild (scored as 1), moderate (scored as 2), and severe (scored as 3). Abbreviation: M; male, F; female, FSGS; focal segmental glomerulosclerosis.

Supplementary Table 2. Small-interfering RNA sequences

Number	Gene	Anti-sense
	**Human**	
1	siEPRS1	CUAAUUCCUCAGCAAGUAU
2	siEPRS1	CCAACCCUUUAUCGCUGCA

Supplementary Table 3. List of primers used for qPCR

Gene	Forward Primer	Reverse Primer
**Mouse**		
Col1a1	TCATCGTGGCTTCTCTGGTC	GACCGTTGAGTCCGTCTTTG
Col3	GACCAAAAGGTGATGCTGGACAG	CAAGACCTCGTGCTCCAGTTAG
Col4	ATGGCTTGCCTGGAGAGATAGG	TGGTTGCCCTTTGAGTCCTGGA
Fn	TGGTGGCCACTAAATACGAA	GGAGGGCTAACATTCTCCAG
Acta2	GGCTCTGGGCTCTGTAAGG	CTCTTGCTCTGGGCTTCATC
Tgf-β	CTCCCGTGGCTTCTAGTGC	GCCTTAGTTTGGACAGGATCTG
Eprs1	AAGCGGAAAAGGCTCCTAAG	CCCAGTCTTTTCTTTATACTCAGCTT
Gars1	GGAGGCAGCACTTTATCCAAG	TCGGAAGCACTCTCCGTTCT
ATP synthase	TCCATCAAAAACATCCAGAAAA	GAGGAGTGAATAGCACCACAAA
Nd1	ATCCTCCCAGGATTTGGAAT	ACCGGTAGGAATTGCGATAA
Rpl13a	CGATAGTGCATCTTGGCCTTT	CCTGCTGCTCTCAAGGTTGTT
18S rRNA	TTCGGAACTGAGGCCATGATT	TTTCGCTCTGGTCCGTCTTG
Ctgf	TGCGAAGCTGACCTGGAGGAAA	CCGCAGAACTTAGCCCTGTATG
Fap	CCGCGTAACACAGGATTCACTG	CACACTTCTTGCTCGGAGGAGA
Gapdh	CATCACTGCCACCCAGAAGACTG	ATGCCAGTGAGCTTCCCGTTCAG
**Human**		
EPRS1	AGGAAAGACCAACACCTTCTC	CTCCTTGAACAGCCACTCTATT
COL1A1	CCTGGCCCCATTGGTAATGTT	CCCCCTCACGTCCAGATTCAC
ACTA2	GATCTGGCACCACTCTTTCTAC	CAGGCAACTCGTAACTCTTCTC
FN	CAAGCCAGATGTCAGAAGC	GGATGGTGCATCAATGGCA
GAPDH	ATCAAGAAGGTGGTGAAGCAG	GTCGCTGTTGAAGTCAGAGG
**Rat**		
Eprs1	TGTCTTCGTGTGATGTGCTG	CAAGAAAGCCATACCAGCGG
Gapdh	CCATCAACGACCCCTTCATT	CACGACATACTCAGCACCAGC

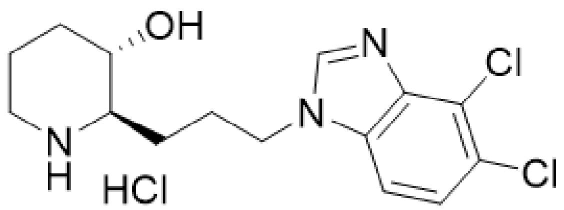

DWN12088

(2R, 3S)-2-(3-(4,5-dichloro-1H-
benzo[d]imidazol-1-yl)propyl) piperidin-3-ol HCl

Supplementary Fig. 1 Chemical structure of DWN12088. (2R, 3S)-2-(3-(4,5-dichloro-1H-benzo[d]imidazol-1-yl)propyl) piperidin-3-ol HCl.

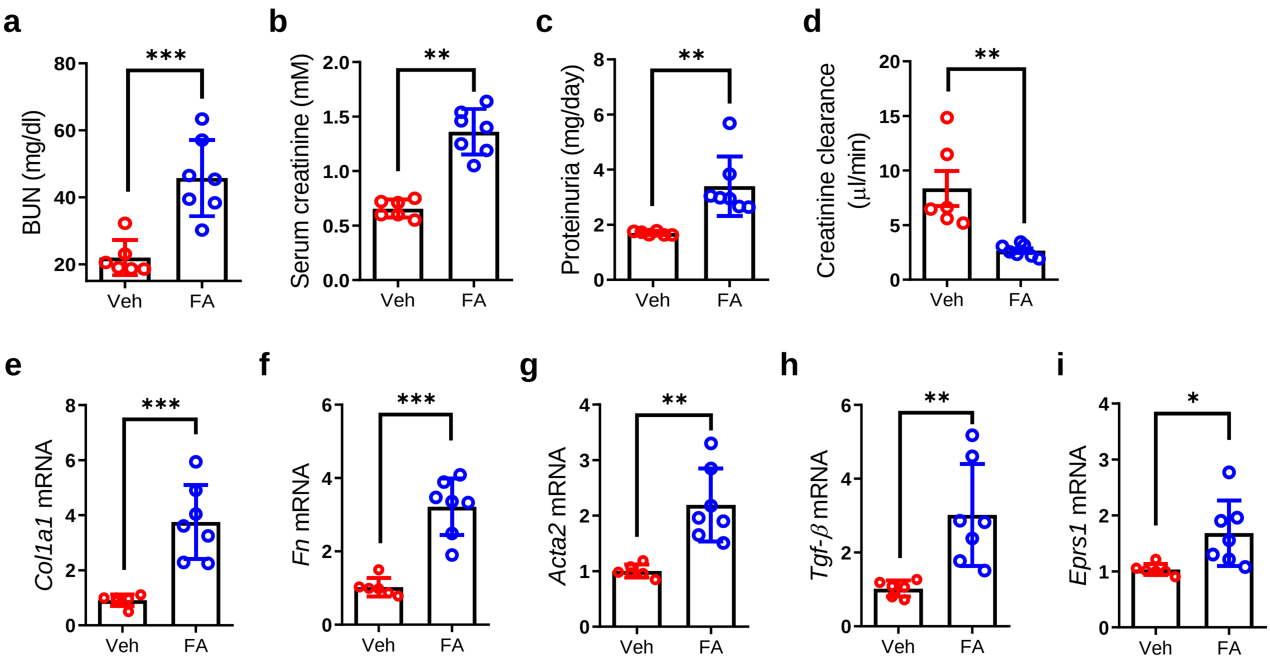

Supplementary Fig. 2 Clinical index and mRNA expression levels in FA mice. a-d Folic acid (FA) induced blood urea nitrogen (BUN), serum creatinine, proteinuria, and creatinine clearance in two groups as indicated (n = 6-7). **e-i** Quantitative analysis of *Col1a1*, *Fn*, *Acta2*, *Tgf-β,* and *Eprs1* mRNA expression by quantitative polymerase chain reaction (qPCR) and normalized to *Rpl13a* (n = 6-7). Data are presented as mean ± standard deviation. Statistical data were analyzed by a two-tailed t-test. * $P < 0.05$, ** $P < 0.01$, and *** $P < 0.001$. Veh, Vehicle.

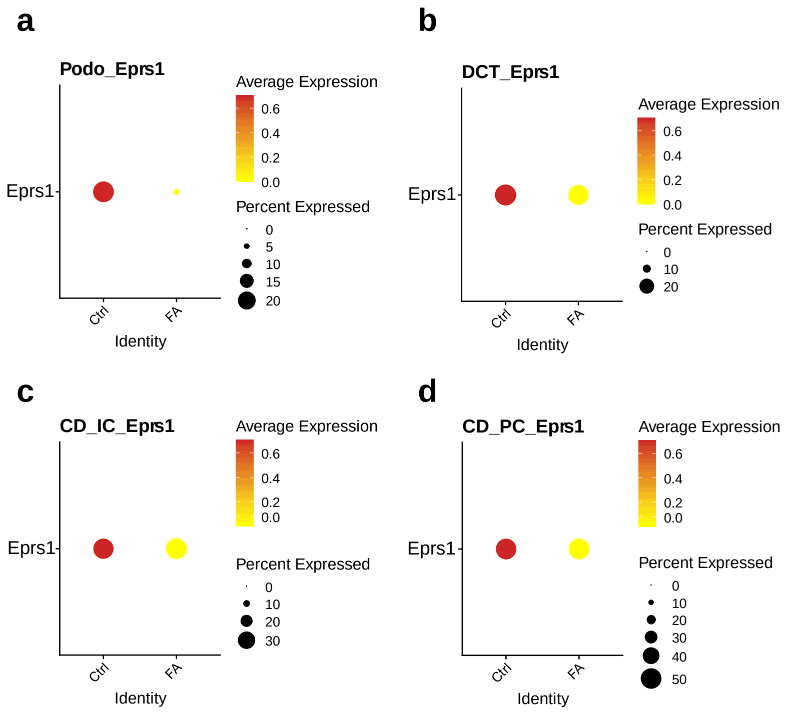

Supplementary Fig. 3 Changes in *Eprs1* genes after subclustering cells in FA mice compared to Veh (Ctrl) mice. a Podocyte. **b** Distal convoluted tubule. **c** Intercalated cells of the collecting duct. **d** Principal cells of the collecting duct.

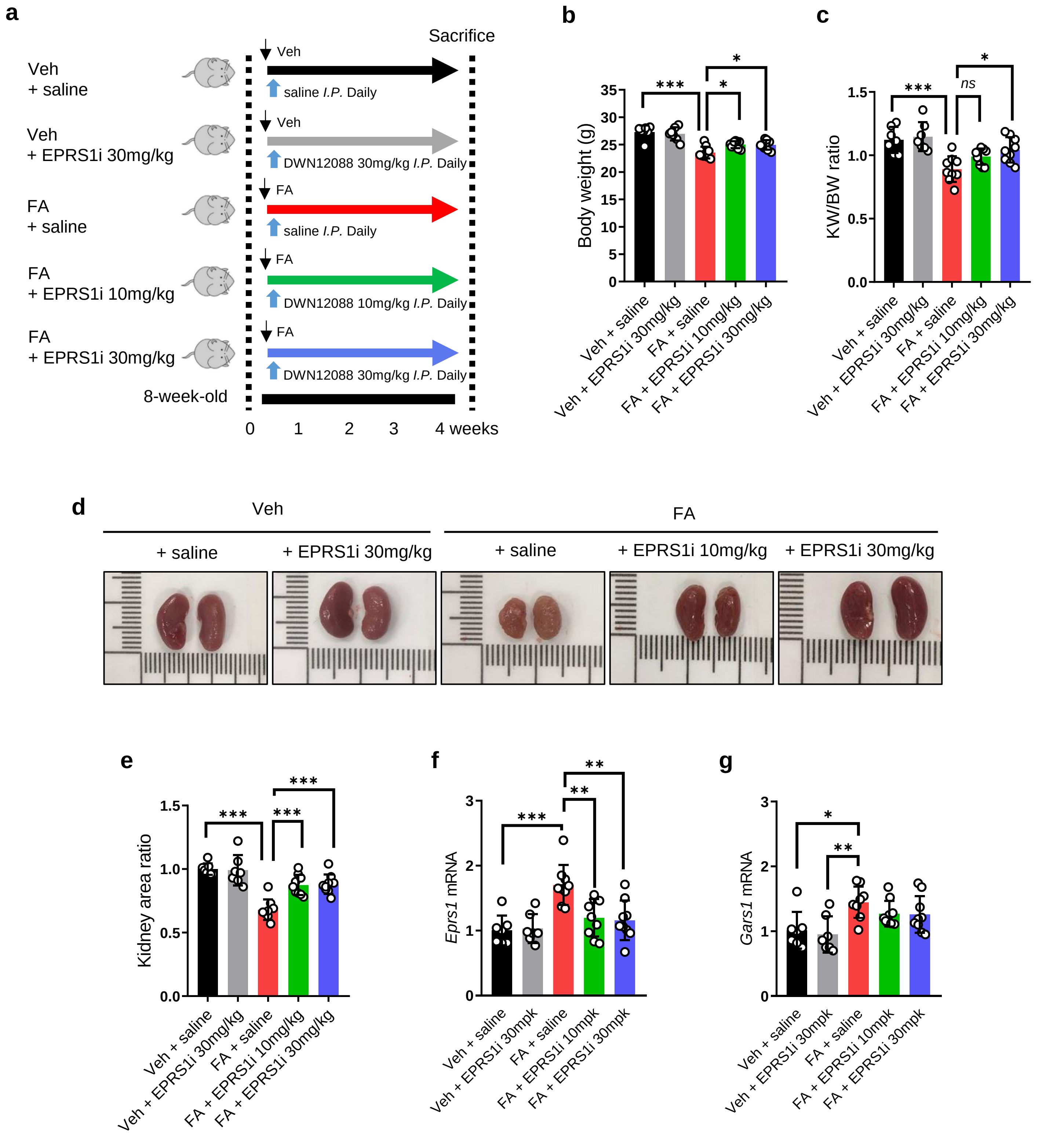

Supplementary Fig. 4 Basic data on effects of pharmacological EPRS1 inhibition in FA mice. a Study design for examining the effect of the EPRS1 inhibitor (EPRS1i) in FA mice (n = 7-9). **b, c** Body weight at 28 days and ratio of kidney weight (KW)-to-body weight (BW) (n = 7-9). **d, e** Gross anatomy of kidney and kidney area ratio measured using ImageJ (n = 7-9). To quantify area in ImageJ, first set the scale. Then, select the region of interest using the selection tools. Finally, measure the area. **f**, **g** mRNA levels of *Eprs1* and *Gars1* were analyzed by qPCR and normalized to *Gapdh* to assess the effect of the EPRS1 inhibitor (n = 7-9). Data are presented as mean ± standard deviation. Statistical data were analyzed by ANOVA with Tukey post-hoc test. * $P < 0.05$, ** $P < 0.01$, and *** $P < 0.001$.

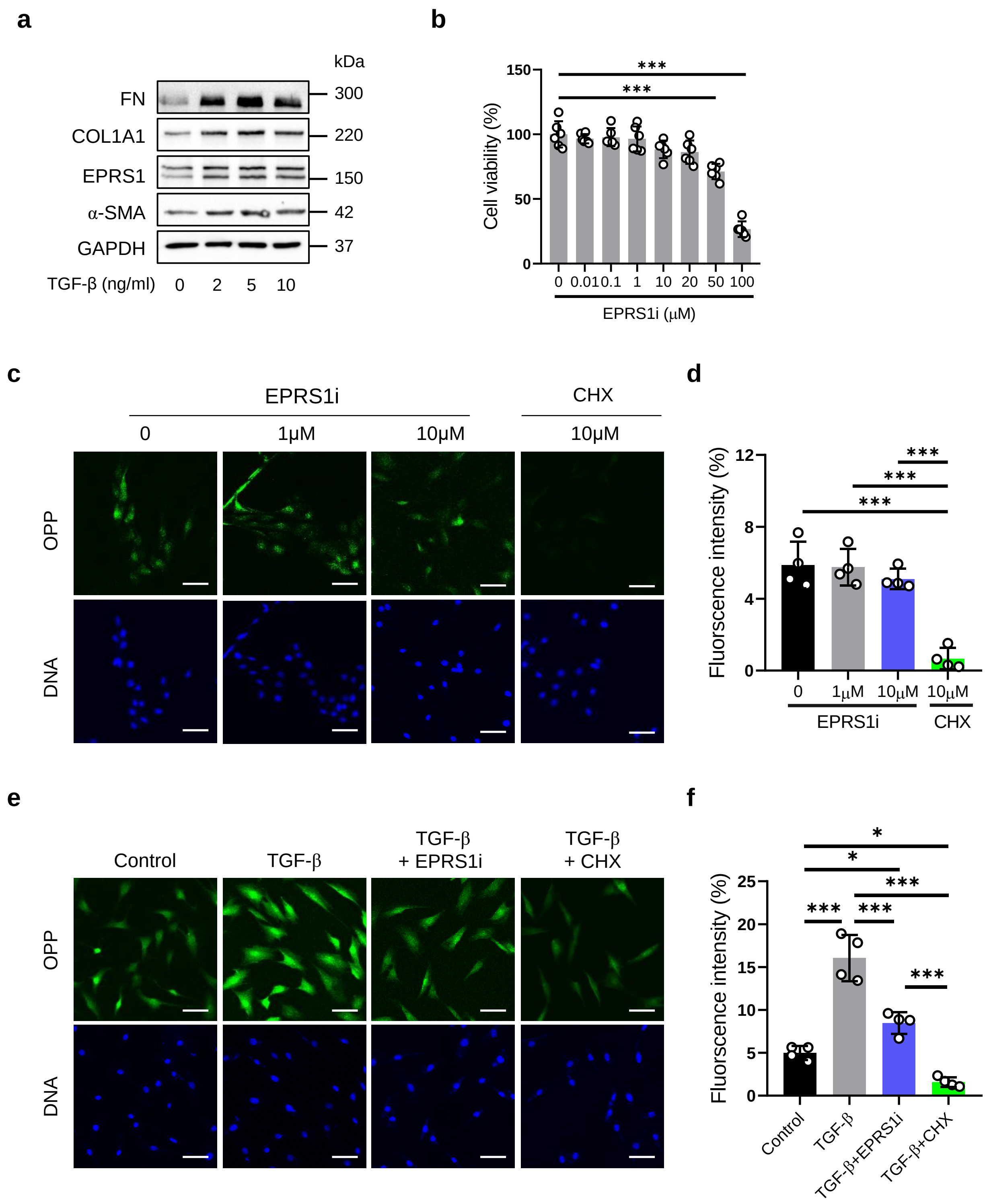

Supplementary Fig. 5 Western blot analysis of fibrosis markers and efficacy of protein translational levels in NRK-49F cells. a Expression of FN, COL1A1, EPRS1, and α-SMA in the whole lysate of NRK-49F cells after 24 h of different treatments by Western blot (*n* = 3). **b** Viability of cells treated with indicated concentrations of EPRS1i for 24 h evaluated by WST-1 assay (*n* = 6). **c** Representative confocal images of OPP (green) in NRK-49F cells treated with EPRS1i or cycloheximide (CHX) for 24 h. **d** OPP staining intensity in NRK-49F cells treated with EPRS1i or CHX. **e** Representative confocal images of OPP (green) in TGF-β induced NRK-49F cells treated with EPRS1i 10μM or CHX 10μM for 24 h. **f** OPP staining intensity in TGF-β induced NRK-49F cells treated with EPRS1i or CHX. * $P < 0.05$ and *** $P < 0.001$. Scale bars = 50μm.

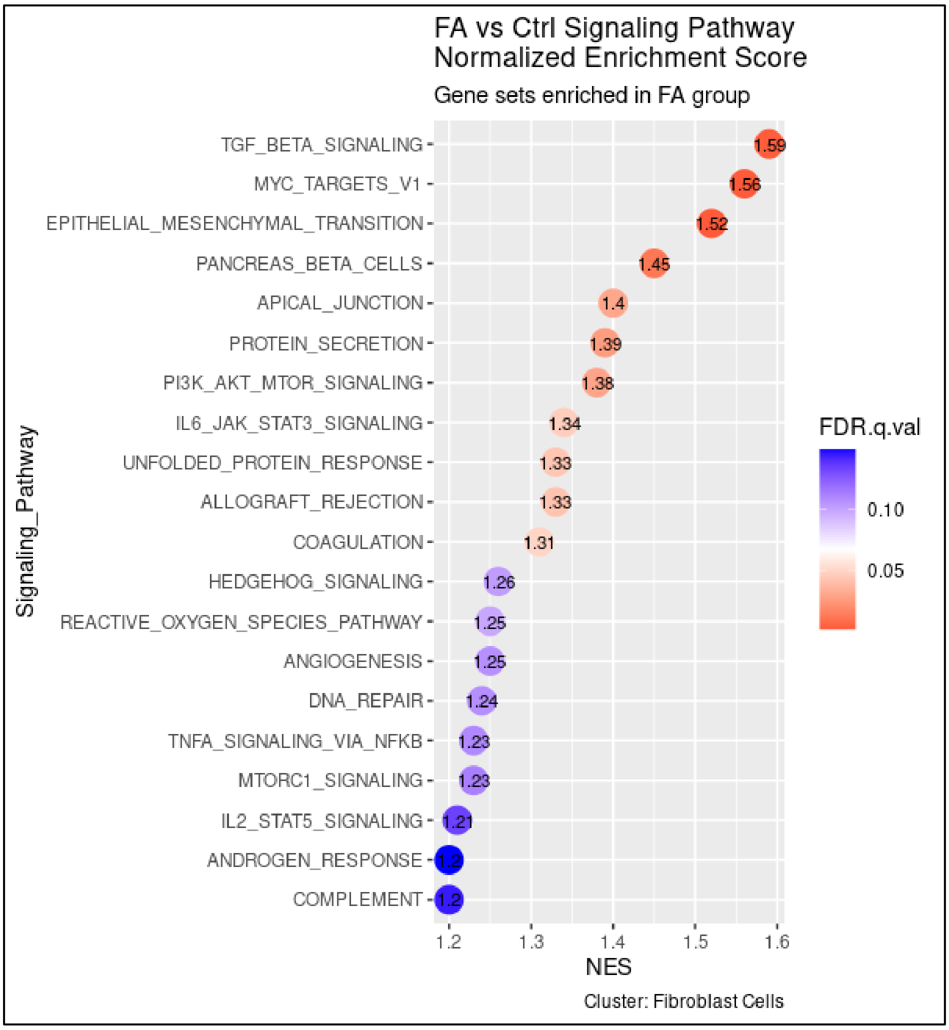

Supplementary Fig. 6 Upregulated signaling pathway in FA compared to control. a Normalized enrichment score for the indicated pathways that are up-regulated in FA compared to control (Ctrl) in total single-cell data.

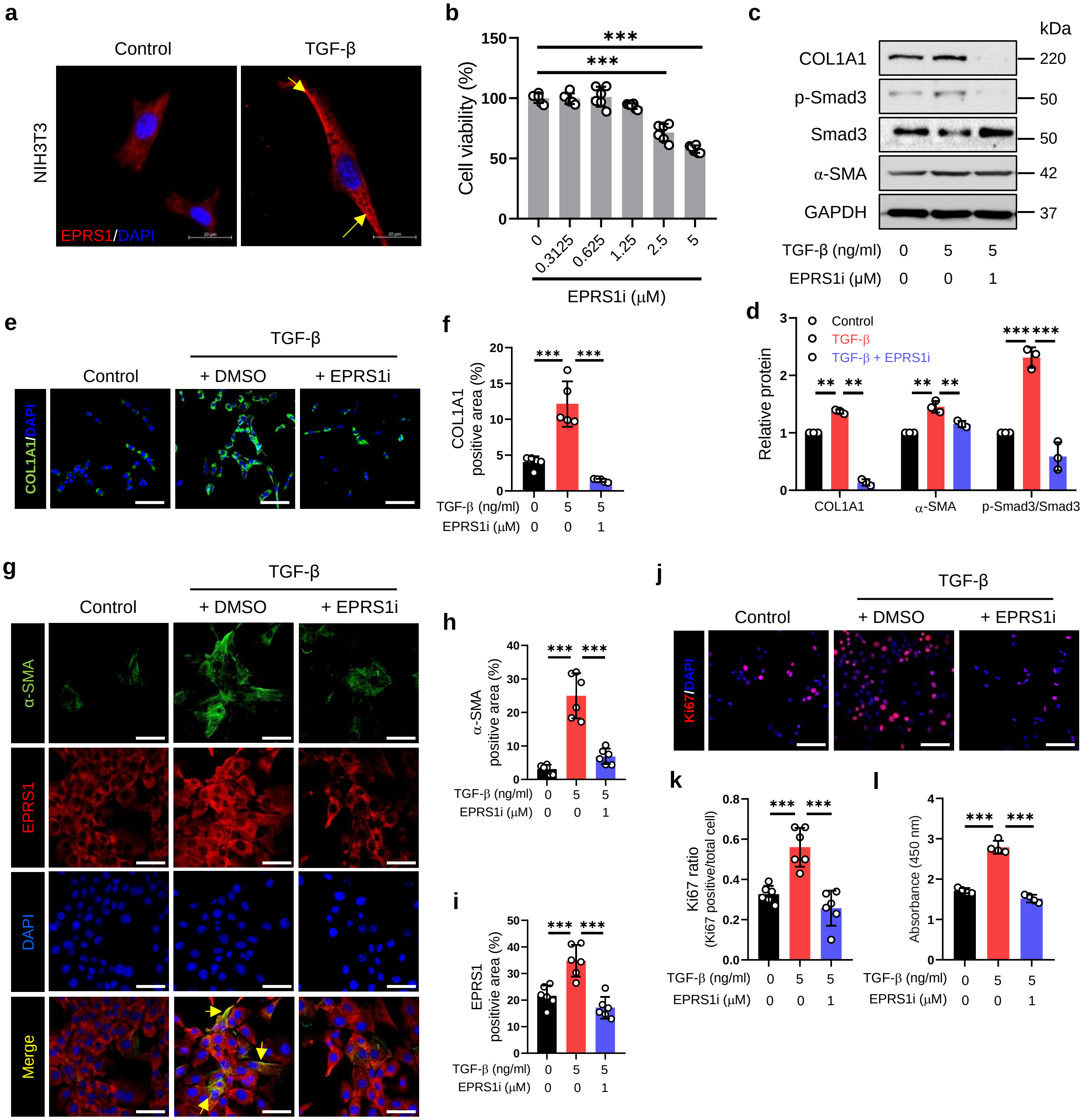

Supplementary Fig. 7 EPRS1 inhibitor suppressed fibroblast activation and proliferation in NIH3T3 cells. a Representative confocal images of EPRS1 (red) in TGF-β (5 ng/ml)-induced NIH3T3 cells for 24 h. Yellow arrow is plasma membrane portion. Scale bar = 20 μm. **b** Cells were treated with various concentrations of EPRS1i, cell viability was determined by WST-1 assay (n = 6). **c, d** Representative Western blot and quantitative data of fibrosis marker protein (COL1A1, and α-SMA) and SMAD associated proteins (p-Smad3, and Smad3) expression in EPRS1i-treated TGF-β (5 ng/ml) conditions for 24 h (n = 3). **e-i** Representative confocal images of α-SMA, EPRS1, and COL1A1 in EPRS1i-treated TGF-β (5 ng/ml) conditions for 24 h. Yellow arrow is co-localization portion. COL1A1 positive area, α-SMA positive area, and EPRS1 positive area were quantified by ImageJ analysis (n = 6). Scale bar of COL1A1 is 50 μm. Scale bar of α-SMA and EPRS1 is 100 μm. **j** Representative confocal images of Ki67 in EPRS1i-treated TGF-β (5 ng/ml) conditions for 24 h. **k** The Ki67 was quantified by ImageJ analysis (n = 5). Scale bar = 50 μm. **l** Cells were treated with EPRS1i in TGF-β (5 ng/ml) conditions for 72 h, cell proliferation was determined by WST-1 assay. Data are presented as mean ± standard deviation. Statistical data were analyzed by ANOVA with Tukey post-hoc test. * $P < 0.05$, ** $P < 0.01$, and *** $P < 0.001$.

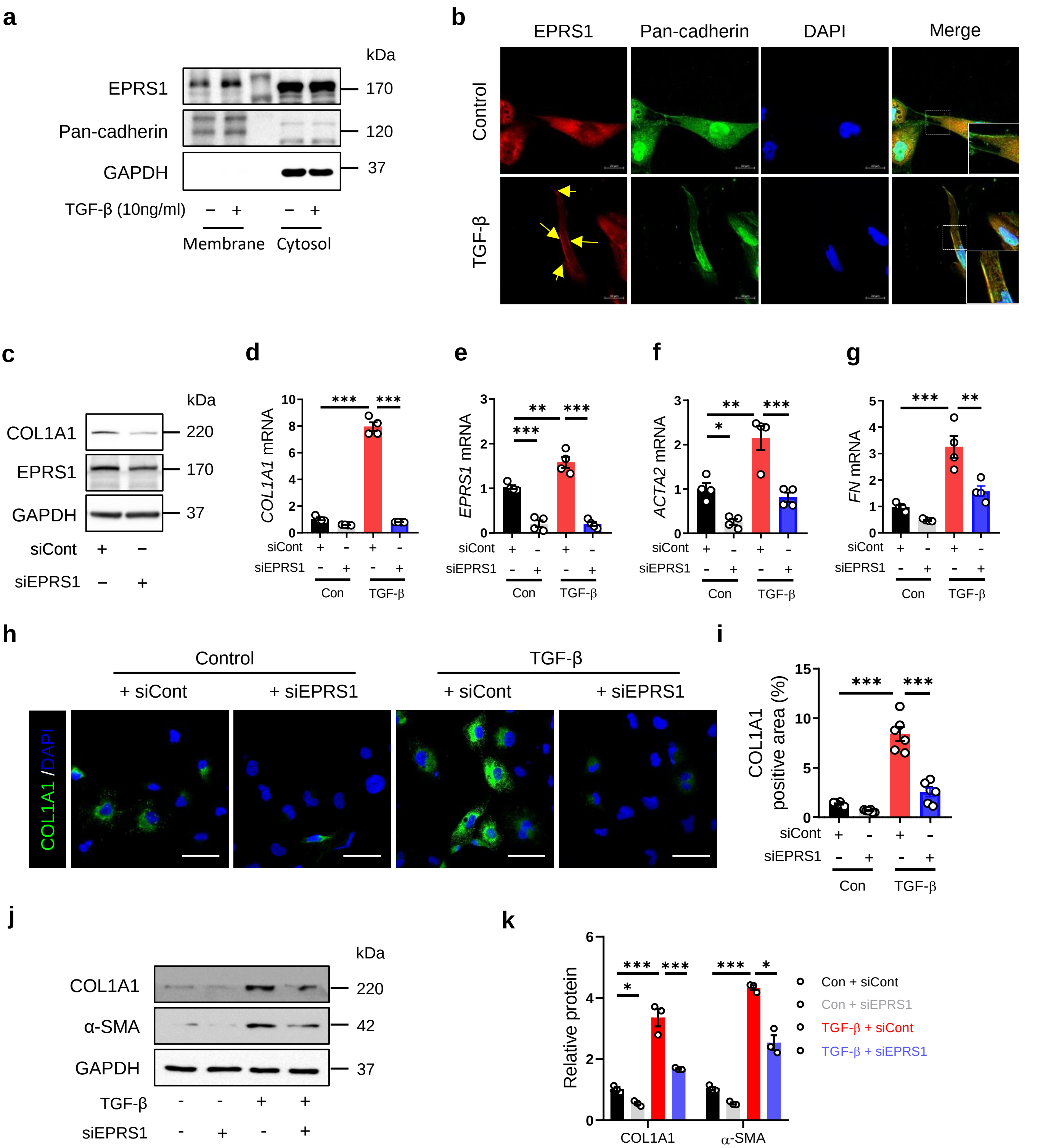

Supplementary Fig. 8 EPRS1 expression in membrane and genetic *EPRS1* inhibition decrease fibrosis markers in HK-2 cells. a Membrane and cytosol fractions of HK-2 cells incubated in the absence or presence of TGF-β for 24 h **b.** Representative confocal images of EPRS1 (red) and Pan-cadherin (green) in TGF-β (5 ng/ml)-induced HK-2 cells for 24 h. The yellow arrow indicates colocalization. **c** Expression of COL1A1 and EPRS1 was determined by Western blot after 24 h of different groups in siRNA-mediated EPRS1 (siEPRS1)-treated HK-2 cells. **d-g** Representative data show mRNA expression levels of *EPRS1* and fibrosis markers (*COL1A1*, *ACTA2*, and *FN*) in siEPRS1 (20 nM)-treated TGF-β (10 ng/ml) conditions. Gene expression levels were analyzed by qPCR and normalized to *GAPDH* (n = 4). **h** Representative confocal images of COL1A1 in siEPRS1 (20 nM)-treated TGF-β (10 ng/ml) conditions. Scale bars = 50 μm. **i** COL1A1 positive area was quantified by ImageJ analysis (n = 6). **j, k** Representative Western blot and quantitative data of fibrosis marker protein (COL1A1, and α-SMA) expression in siEPRS1 (20 nM)-treated TGF-β (10 ng/ml) conditions (n = 3). Data are presented as mean ± standard deviation. Statistical data were analyzed by ANOVA with Tukey post-hoc test. * $P < 0.05$, ** $P < 0.01$, and *** $P < 0.001$.

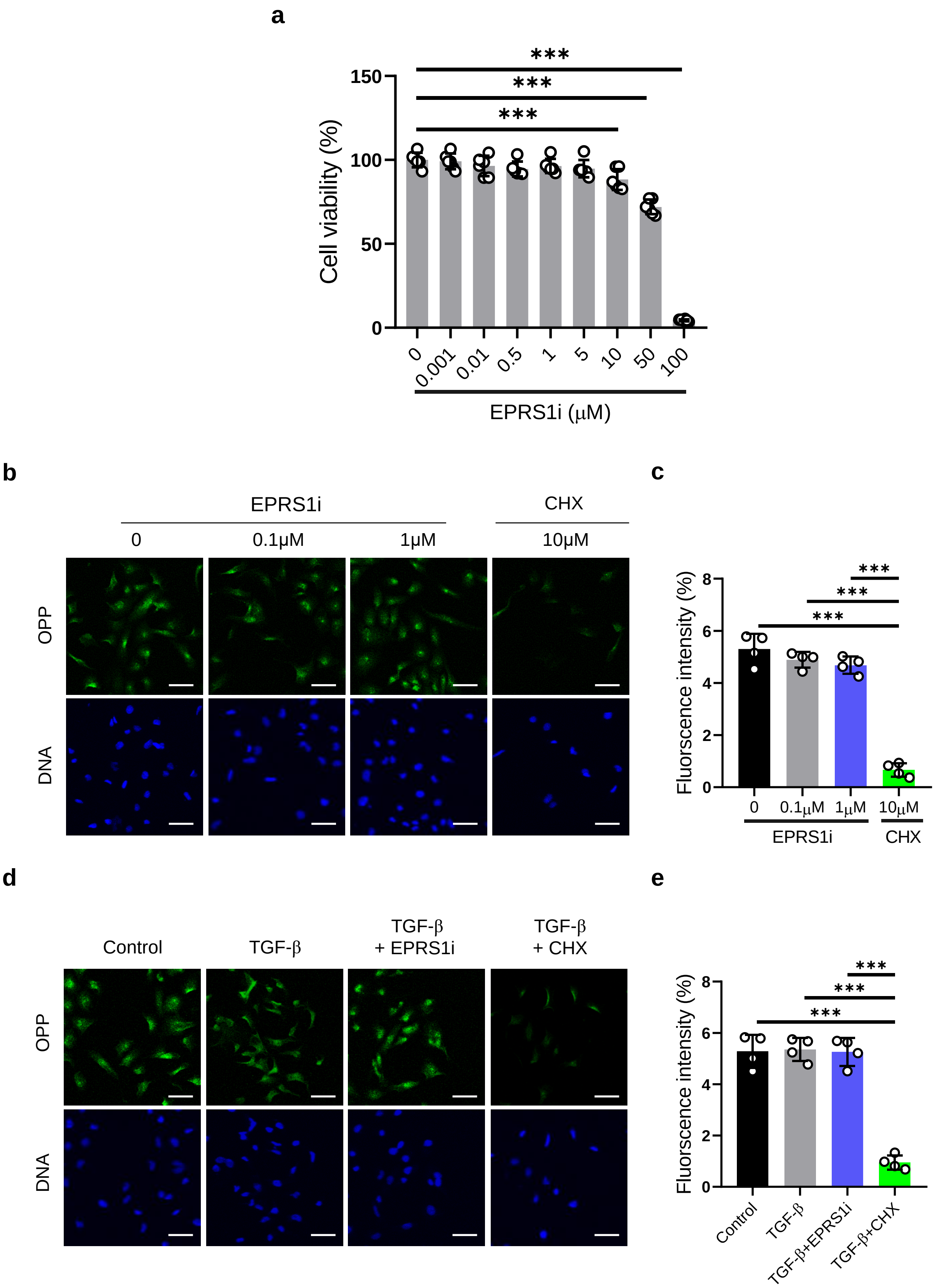

Supplementary Fig. 9 Cell viability and efficacy of protein translational levels in HK-2 cells. a HK-2 cells were treated with various concentrations of EPRS1i, cell viability was determined by WST-1 assay (n = 6). **b** Representative confocal images of OPP (green) in HK-2 cells treated with EPRS1i or CHX for 24 h. **c** OPP staining intensity in HK-2 cells treated with EPRS1i or CHX. **d** Representative confocal image of OPP (green) in TGF-β (10 ng/ml) induced HK-2 cells treated with EPRS1i 1μM or CHX 10μM for 24 h. **e** OPP staining intensity in TGF-β induced HK-2 cells treated with EPRS1i or CHX. Data are presented as mean ± standard deviation. Statistical data were analyzed by ANOVA with Tukey post-hoc test. *** P < 0.001. Scale bars = 50μm.

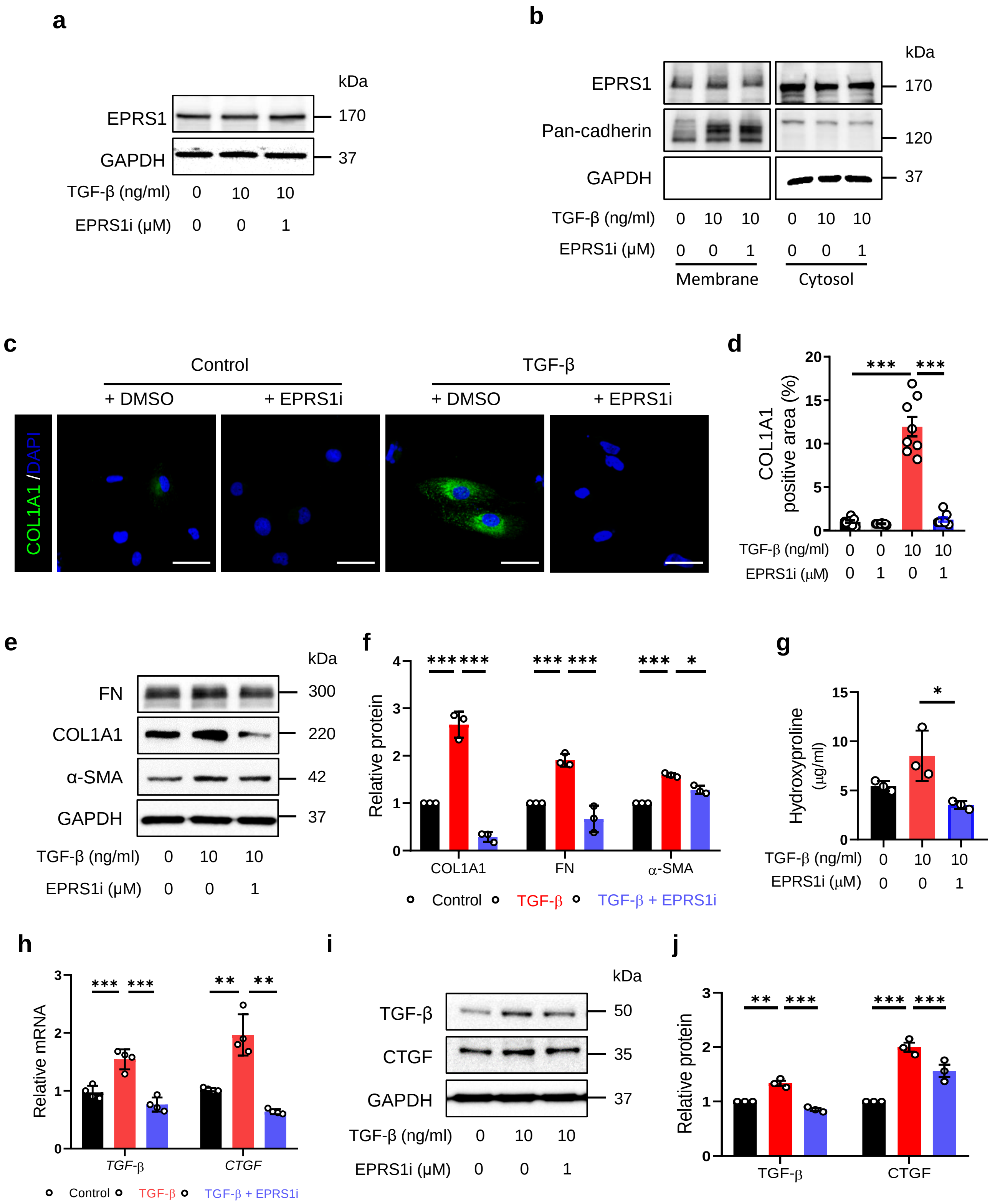

Supplementary Fig. 10 Pharmacological EPRS1 inhibition decreases EPRS1 translocation and fibrosis markers in HK-2 cells. **a** The protein expression of EPRS1 in whole lysate under EPRS1i-treated TGF-β (10 ng/ml) conditions for 24 h. **b** Membrane and cytosol fractions of EPRS1i-treated HK-2 cells incubated in the absence or presence of TGF-β for 24 h. **c, d** Representative micrographs and quantitative data show COL1A1 in EPRS1i-treated TGF-β (10 ng/ml) conditions for 24 h (n = 8). Scale bars = 50 μm. **e**, **f** Representative Western blot and quantitative data of fibrosis marker proteins (FN, COL1A1, and α-SMA) in EPRS1i-treated TGF-β (10 ng/ml) conditions for 24 h (n = 3). **g** Levels of hydroxyproline in cultured cells were quantified using ELISA (n = 3). **h** Relative mRNA levels of profibrotic marker genes (*TGF-β* and *CTGF*) were analyzed by qPCR and normalized to *GAPDH* (n = 4). **i, j** Representative Western blot and quantitative data showing effects of EPRS1i on profibrotic marker proteins (TGF-β and CTGF) in EPRS1i-treated TGF-β (10 ng/ml) conditions for 24 h (n = 3). Data are presented as mean ± standard deviation. Statistical data were analyzed by ANOVA with Tukey post-hoc test. * $P < 0.05$, ** $P < 0.01$, and *** $P < 0.001$.

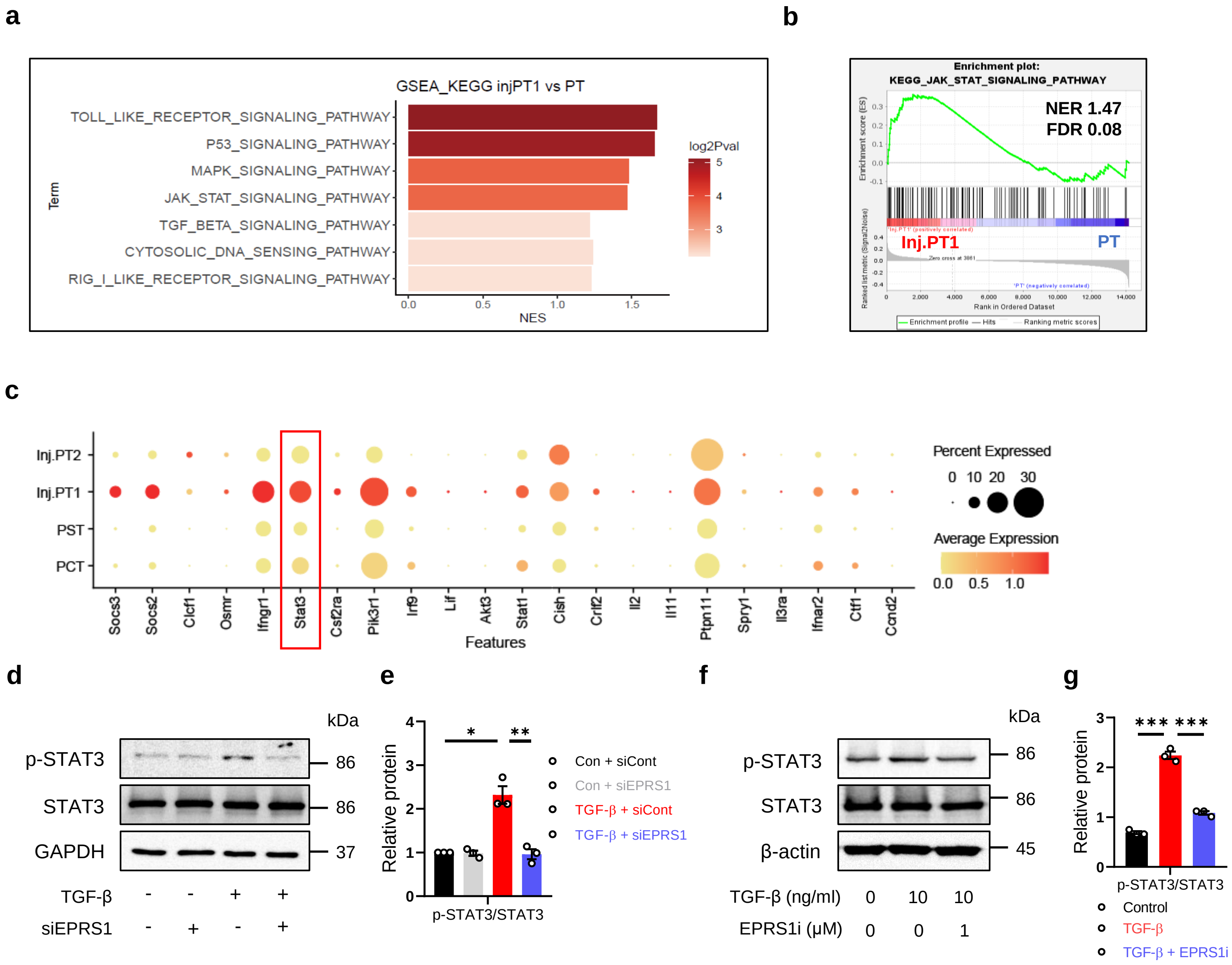

Supplementary Fig. 11 The EPRS1 inhibition decreases p-STAT3 expression in proximal tubules. a KEGG pathway of Inj. PT1 and PT (PST + PCT). **b** Gene Set Enrichment Analysis (GSEA) software plots for the indicated pathways that are up-regulated in Inj. PT1 cells compared to PT cells in total single-cell data. **c** The gene expression features show that differentially expressed genes (DEGs; FA mice was compared with Vehicle mice) in every cell type. **d, e** Representative Western blot and quantitative data of STAT3 associated proteins (p-STAT3 and STAT3) expression in siEPRS1 (20 nM)-treated TGF-β (10 ng/ml) conditions for 24 h (*n* = 3). **f, g** Representative Western blot and quantitative data of STAT3 associated proteins (p-STAT3 and STAT3) expression in EPRS1i-treated TGF-β (10 ng/ml) conditions for 24 h (*n* = 3). Data are presented as mean ± standard deviation. Statistical data were analyzed by ANOVA with Tukey post-hoc test. * $P < 0.05$, ** $P < 0.01$, and *** $P < 0.001$.

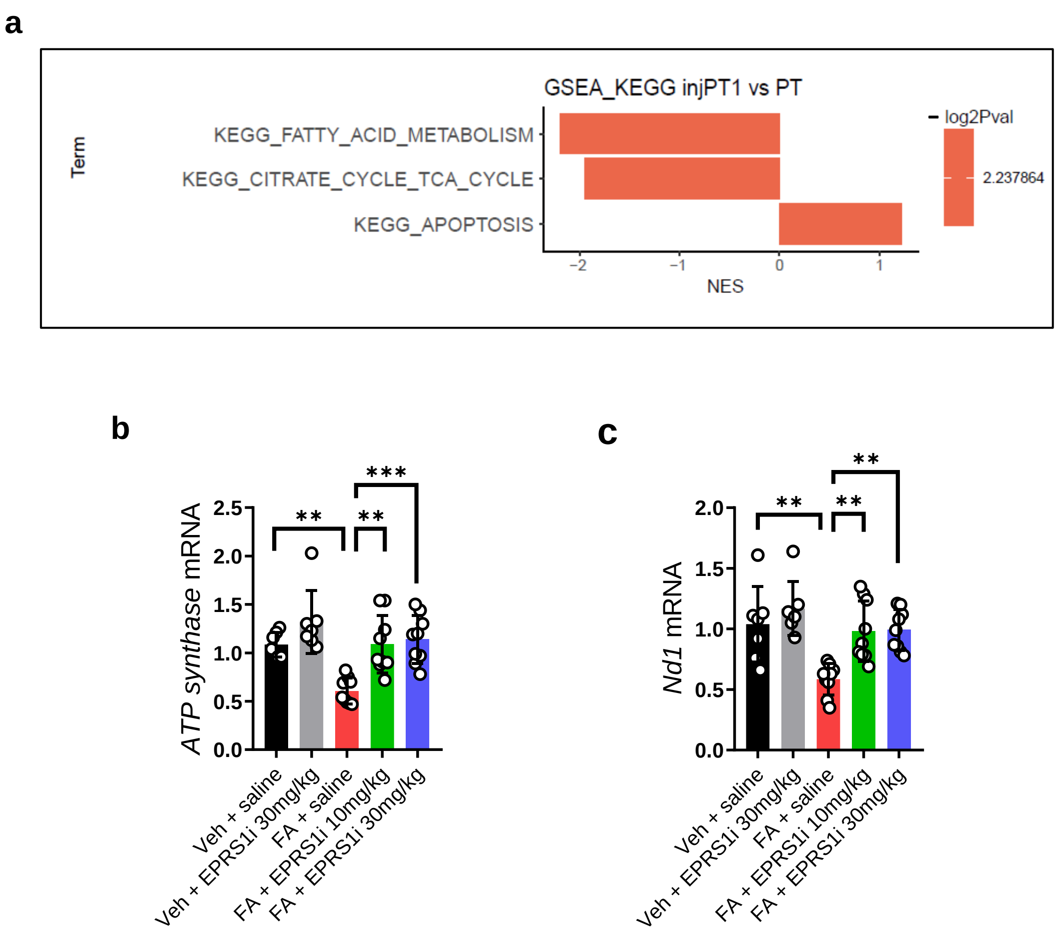

Supplementary Fig. 12 Mitochondrial related KEGG pathway in single cell analysis and gene expression of mitochondrial dysfunction markers in EPRS1 inhibitor treated mice. a KEGG pathway of Inj. PT1 and PT (PCT + PST). **b, c** Quantitative analysis of *ATP synthase* and *NADH dehydrogenase 1* (*Nd1)* expression normalized to *18S rRNA* by qPCR (n = 7-9). Data are presented as mean ± standard deviation. Statistical data were analyzed by ANOVA with Tukey post-hoc test. ** $P < 0.01$ and *** $P < 0.001$.

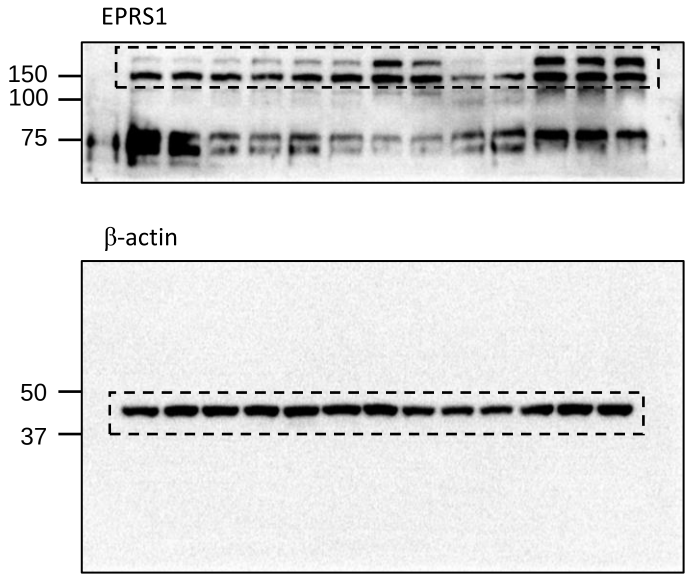

Supplementary Fig. 13 Full blots of Fig. 1

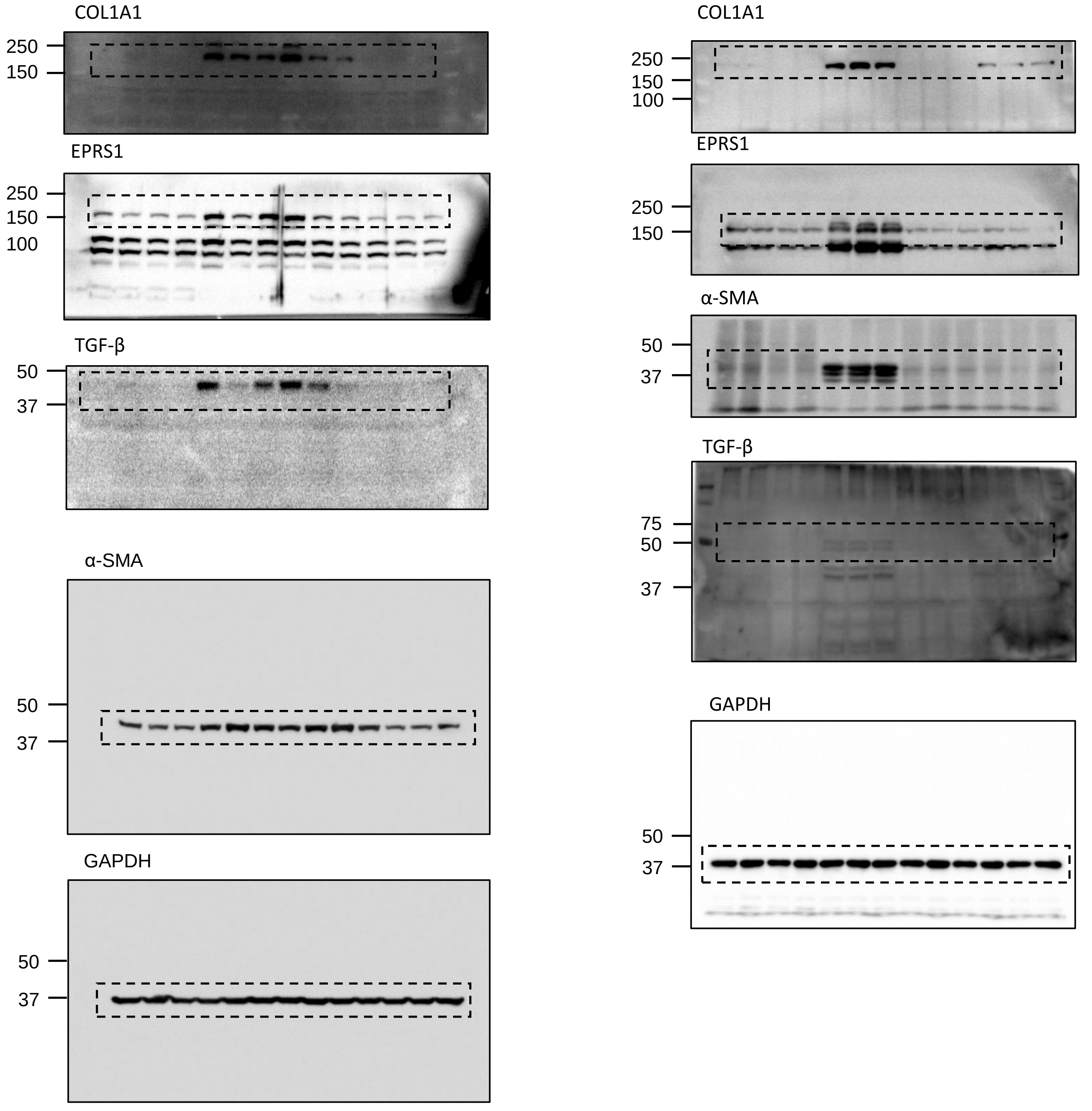

Supplementary Fig. 14 Full blots of Fig. 4

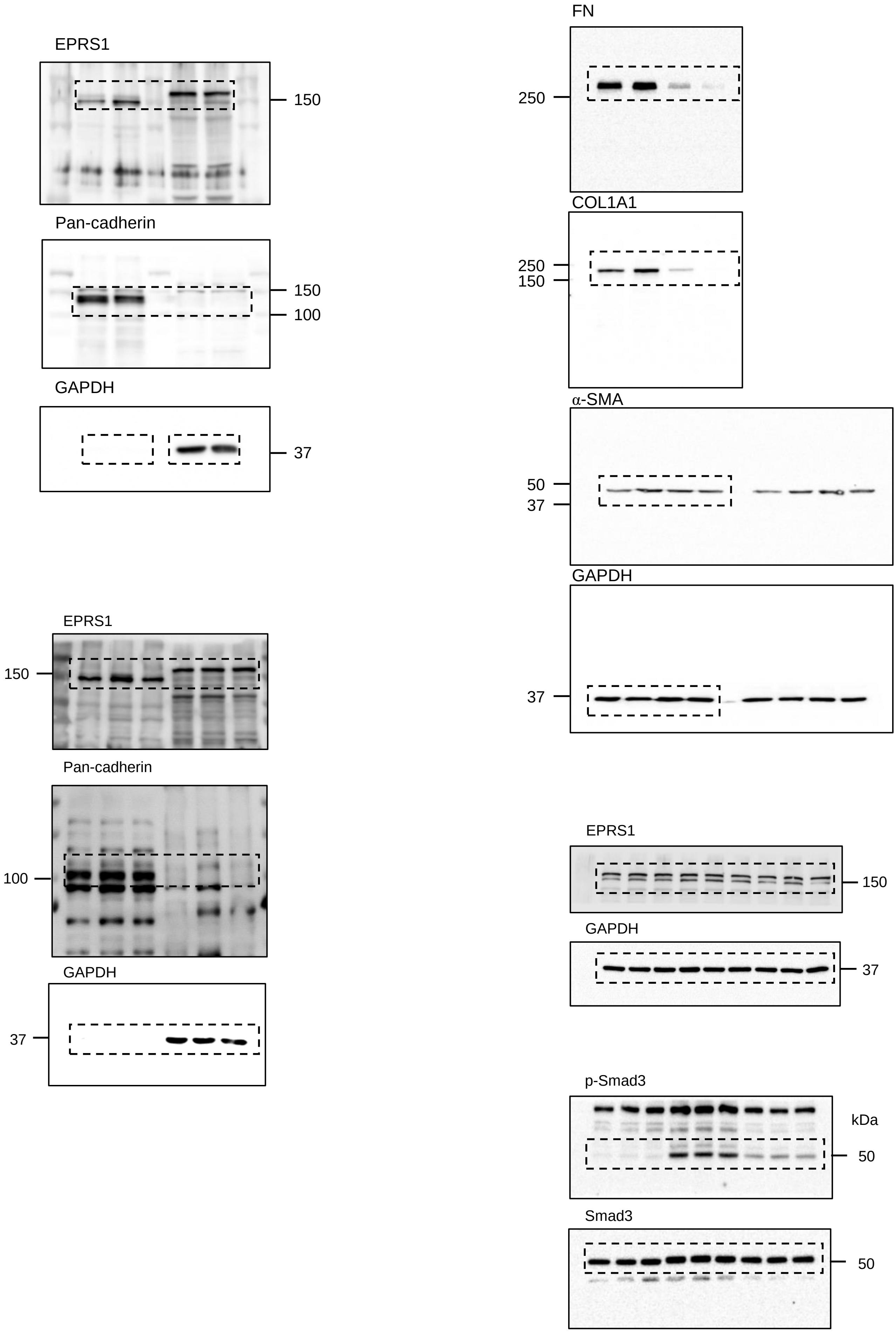

Supplementary Fig. 15 Full blots of Fig. 6

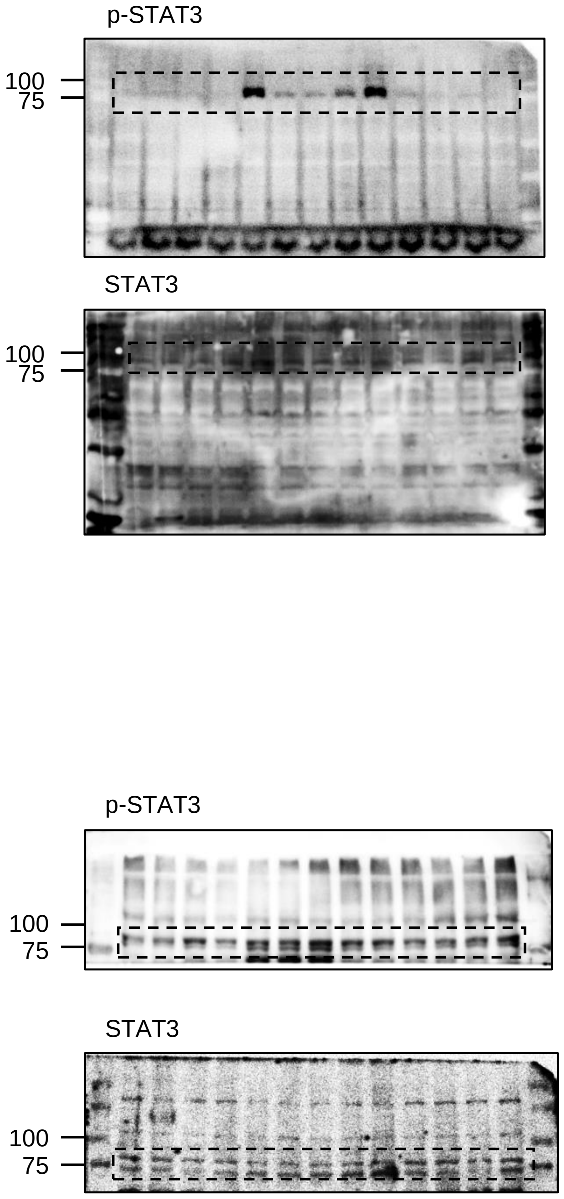

Supplementary Fig. 16 Full blots of Fig. 7

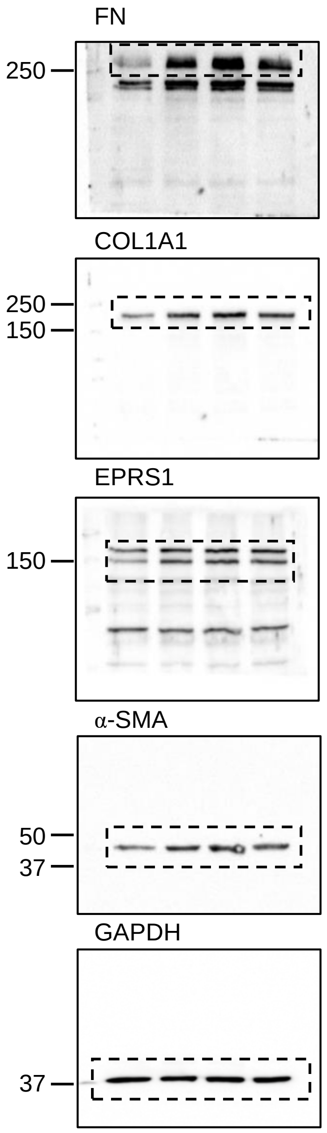

Supplementary Fig. 17 Supplementary Fig. 5

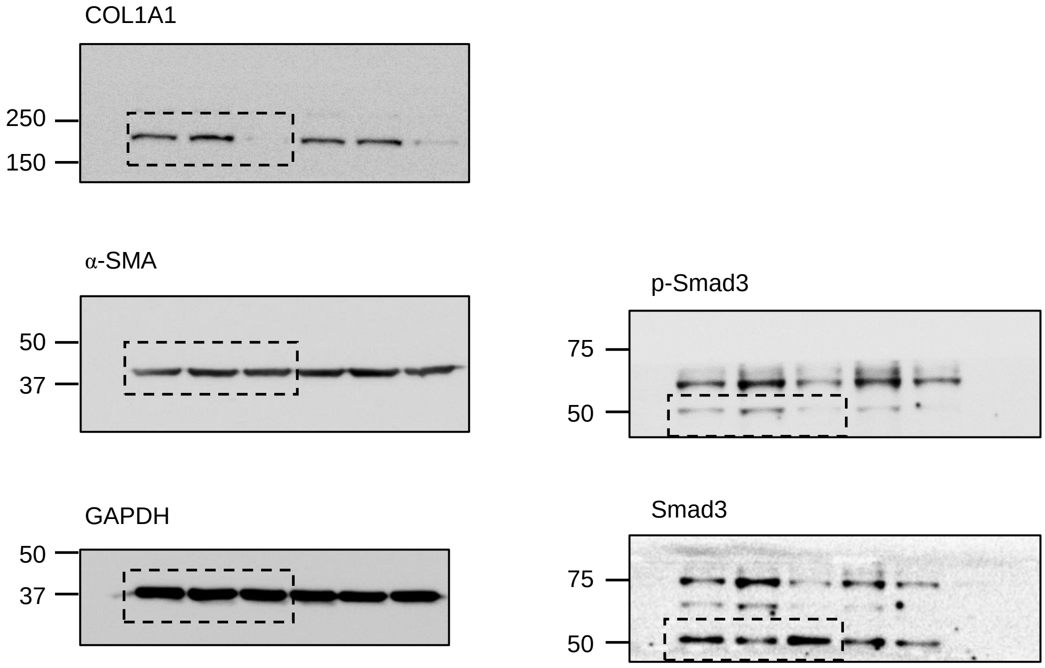

Supplementary Fig. 18 Supplementary Fig. 7

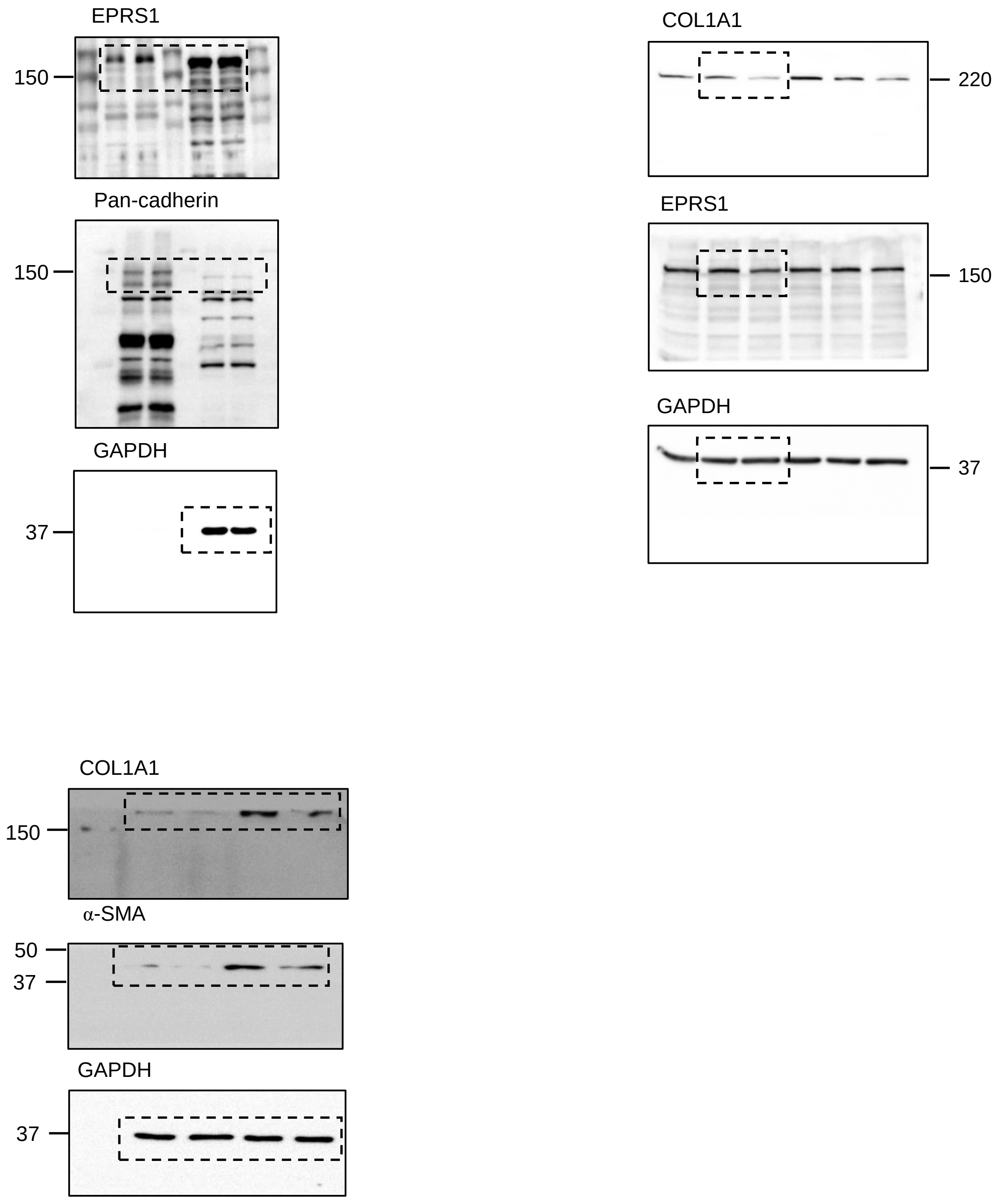

Supplementary Fig. 19 Full blots of Supplementary Fig. 8

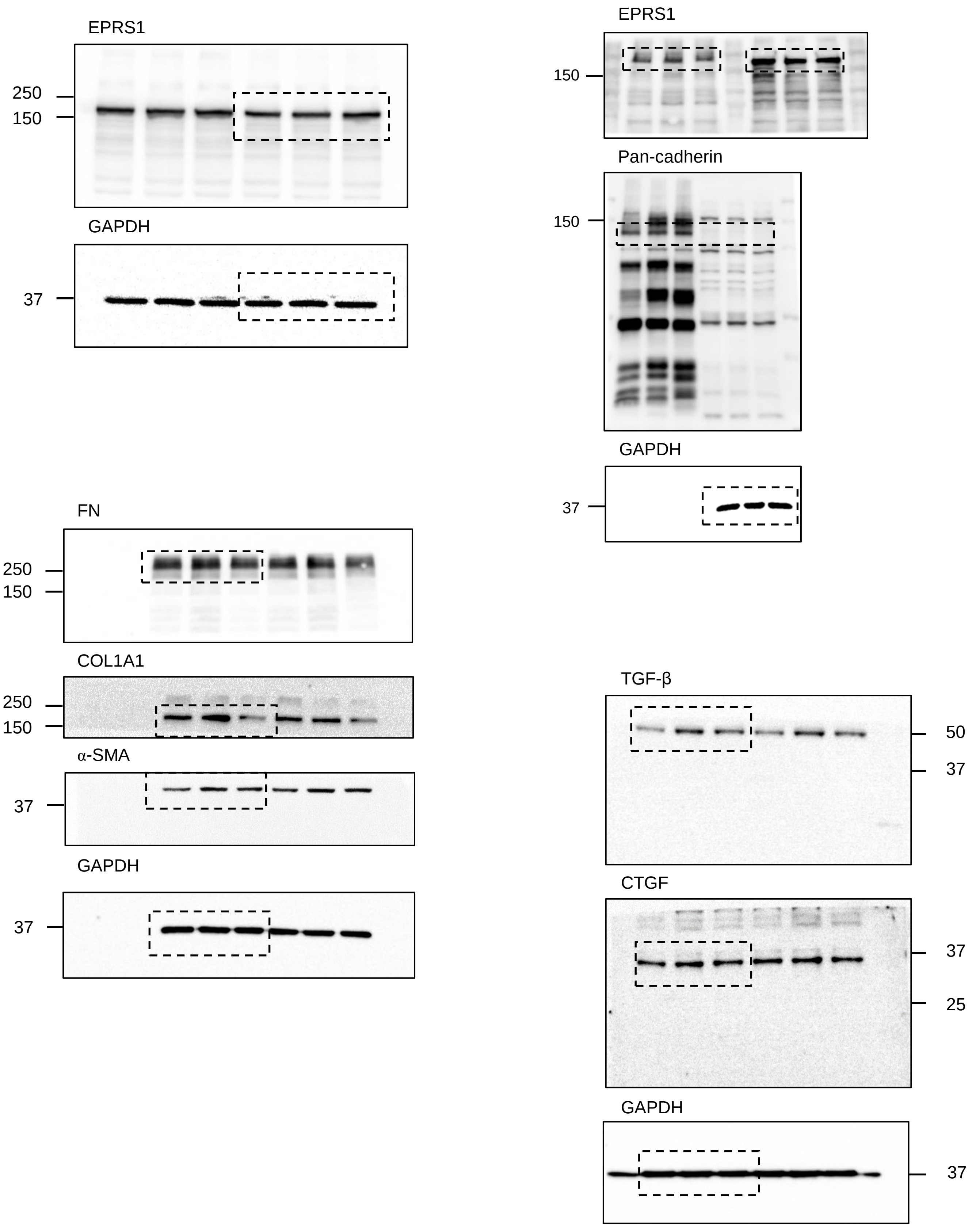

Supplementary Fig. 20 Full blots of Supplementary Fig. 10

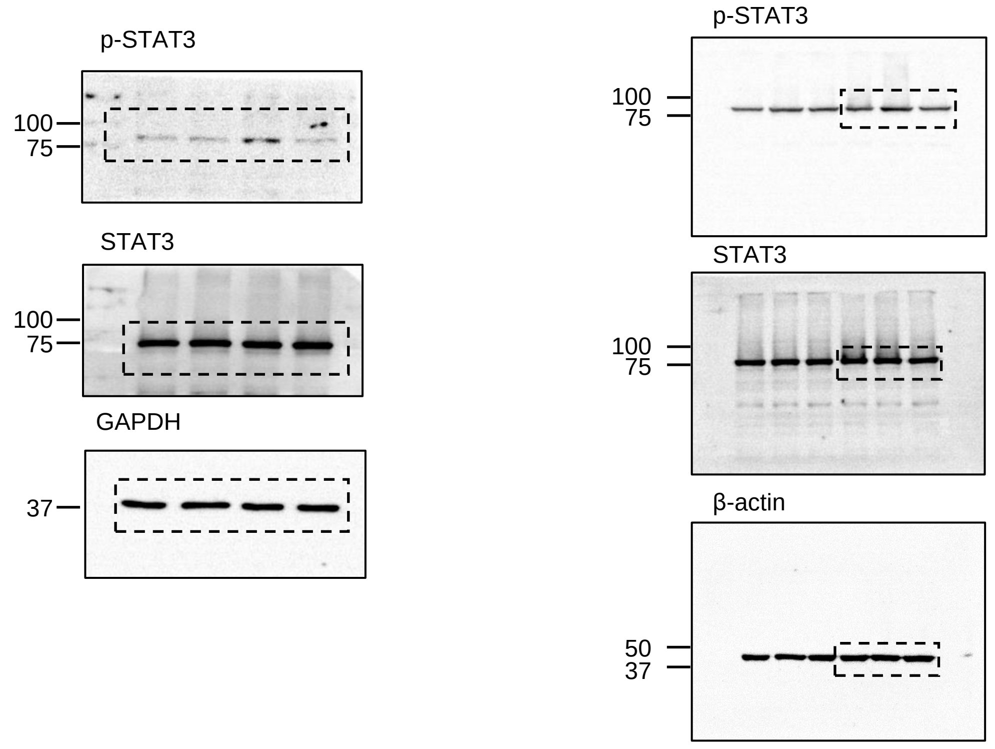

Supplementary Fig. 21 Full blots of Supplementary Fig. 11
